# Tree size diversity is the major driver of aboveground carbon storage in dryland agroforestry parklands

**DOI:** 10.1038/s41598-023-49119-9

**Published:** 2023-12-14

**Authors:** Florent Noulèkoun, Sylvanus Mensah, HyungSub Kim, Heejae Jo, Gérard N. Gouwakinnou, Thierry D. Houéhanou, Michael Mensah, Jesse Naab, Yowhan Son, Asia Khamzina

**Affiliations:** 1 Agroforestry Systems and Ecology Laboratory (ASEL), Department of Environmental Science and Ecological Engineering, Korea University, Seoul, 02841 South Korea; 2 Laboratoire de Biomathématiques et d’Estimations Forestières, Faculté des Sciences Agronomiques, Université d’Abomey Calavi, Cotonou, Benin; 3 Chair of Forest Growth and Dendroecology, Albert-Ludwigs-Universität Freiburg, Freiburg im Breisgau, Germany; 4 Ecosystem Ecology Laboratory, Department of Environmental Science and Ecological Engineering, Korea University, Seoul, 02841 South Korea; 5 Research Unit of Biodiversity Conservation at the Interface People-Land Use and Climate Changes, Laboratory of Ecology, Botany and Plant Biology, Faculty of Agronomy, University of Parakou, BP 125, Parakou, Benin; 6 Department of Business Administration, University of Professional Studies, Accra, Ghana; 7 West African Science Service Center on Climate Change and Adapted Land Use (WASCAL), P.O. Box 9507, Ouagadougou 06, Burkina Faso

**Keywords:** Forestry, Biodiversity

## Abstract

Despite the importance of agroforestry parkland systems for ecosystem and livelihood benefits, evidence on determinants of carbon storage in parklands remains scarce. Here, we assessed the direct and indirect influence of human management (selective harvesting of trees), abiotic factors (climate, topography, and soil) and multiple attributes of species diversity (taxonomic, functional, and structural) on aboveground carbon (AGC) stocks in 51 parklands in drylands of Benin. We used linear mixed-effects regressions and structural equation modeling to test the relative effects of these predictors on AGC stocks. We found that structural diversity (tree size diversity, H_DBH_) had the strongest (effect size β = 0.59, R^2^ = 54%) relationship with AGC stocks, followed by community-weighted mean of maximum height (CWM_MAXH_). Taxonomic diversity had no significant direct relationship with AGC stocks but influenced the latter indirectly through its negative effect on CWM_MAXH_, reflecting the impact of species selection by farmers. Elevation and soil total organic carbon content positively influenced AGC stocks both directly and indirectly via H_DBH_. No significant association was found between AGC stocks and tree harvesting factor. Our results suggest the mass ratio, niche complementarity and environmental favorability as underlying mechanisms of AGC storage in the parklands. Our findings also highlight the potential role of human-driven filtering of local species pool in regulating the effect of biodiversity on AGC storage in the parklands. We conclude that the promotion of AGC stocks in parklands is dependent on protecting tree regeneration in addition to enhancing tree size diversity and managing tall-stature trees.

## Introduction

Agroforestry—land-use systems and technologies that integrate trees, crops and/or animals on the same land unit in some form of temporal sequence or spatial arrangement—is practiced worldwide for improving environmental sustainability of contemporary agriculture and providing multiple ecosystem services including food production, biodiversity conservation and carbon sequestration^1–4^. Because of their potential of sequestering large amounts of atmospheric carbon dioxide, agroforestry systems have been recognized among climate change mitigation strategies under the Kyoto Protocol since 2007^2,5^. Current estimates of the carbon sequestration potential of various agroforestry systems are highly variable, but it could amount up to 2.2. Pg C (1 Pg = 10^15^ g) over 50 years in the combined above- and belowground biomass pools^6^.

Among various types of agroforestry systems practiced in the tropics, parklands represent scattered multipurpose trees maintained in cultivated or recently fallowed cropping fields^7,8^. Agroforestry parklands share ecological characteristics of both forests, and agricultural lands. The practice of parklands involves a strong human-driven filtering of species, whereby farmers select, conserve and manage the tree species that provide food, fodder, medicine and other non-timber forest products to local communities^9–11^. Agroforestry parklands can also substantially contribute to climate change mitigation owing to their carbon sequestration potential^12–14^. For example, Luedeling and Neufeldt^12^ reported that if parklands covered their maximum range in the Sahelian productive land, the carbon stocks there would reach 1.3 Pg. However, we still lack knowledge of the major drivers of biomass carbon storage in these human-managed systems, as most of the existing evidence in the tropics is specific to forests^15,16^ and agroforestry homegardens^17,18^. A better understanding of the determinants of carbon storage in parklands is crucial to optimize their carbon sequestration and conservation potential, in support of nature-based solutions for climate change mitigation.

**Figure 1:**
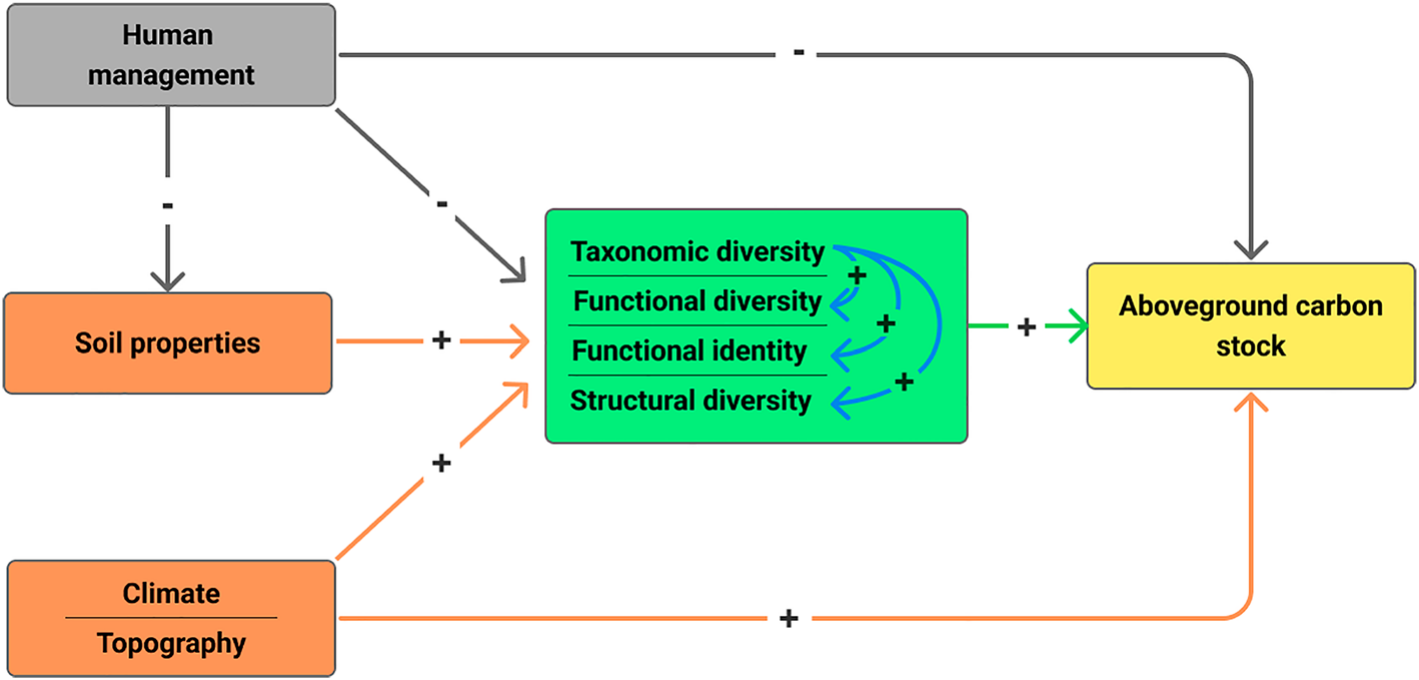
Conceptual model, displaying the hypothesized causal relationships between human management (tree harvesting), abiotic (climate, topography, and soil physical and chemical) and biotic (taxonomic, functional, and structural diversity) factors and aboveground carbon (AGC) stocks. Based on the existing ecological theories and demonstrated relationships between human management, environment, tree community properties and aboveground biomass (AGB) or AGC for forests, we assumed that (i) tree wood harvesting would have a negative effect on soil nutrients, species diversity attributes and AGC stocks, (ii) abiotic conditions that are favorable for tree growth would promote species diversity attributes and AGC stocks, (iii) species diversity attributes positively influence AGC stocks and (iv) taxonomic diversity would enhance functional and structural diversity.

Biotic, abiotic, and human disturbance factors reportedly determined carbon stocks in forests. Regarding the biotic factors, multiple attributes of biodiversity including taxonomic, functional (diversity and identity) and structural ones can directly and indirectly influence aboveground carbon (AGC) stocks (Fig. 1) under the assumptions of the niche complementarity and mass ratio hypotheses^15,16,19–22^. The niche complementarity hypothesis predicts that high functional trait-related niche differentiation or facilitation would enhance ecosystem functioning (e.g., AGC storage) in diverse tree communities due to a more efficient use of resources by co-occurring species^23,24^. The mass ratio hypothesis posits that the functional traits of dominant species would drive AGC stocks within a community^25^. Accordingly, empirical evidence shows that higher taxonomic diversity (e.g., species richness) can directly increase forest AGC stocks through niche complementarity^26,27^. Similarly, a higher functional diversity can promote AGC storage due to complementarity between co-occurring species^28–30^. Alongside the effects of taxonomic and functional diversity, the dominance of functionally important species (i.e., functional identity) can increase AGC stocks due to their disproportionally high contribution to ecosystem functioning^28,31,32^. Finally, structural diversity, defined as the diversity and inequality of tree size in a plant community^33,34^, can have direct and positive effects on AGC, since high structural diversity enhances resource use efficiency^15,35,36^. In addition to the direct effects, taxonomic diversity can have indirect effects on AGC stocks through the other diversity attributes (Fig. 1). For instance, species richness can indirectly increase AGC storage via structural diversity because increasing species richness could lead to a better occupation of available spatial niches by co-occurring species, thereby optimizing the utilization of space and resources^15,19,36^.

Climate and soil conditions (e.g., water and nutrient availability) can also influence AGC storage both directly and indirectly via biotic factors by stimulating processes like competition and facilitation^15,19,37^ (Fig. 1). We expect that higher water availability and soil fertility would increase AGC storage under the assumption that higher resource availability promotes faster plant growth and greater tree species, functional and structural diversity^27,38–40^. In addition, topography can influence plant growth through its impact on local climate and soil conditions, thereby regulating AGC stocks^41–43^. For instance, elevation and slope strongly control local-scale soil chemistry, hydrology, and microclimate, thereby constraining the conditions within which trees grow and shaping the composition and structure of tree communities^42^. Finally, human disturbance such as excessive logging, resulting in forest degradation and fragmentation, can alter stand structures, species demography and the functional composition of tree assemblages, reducing the forest AGC storage^16,44,45^.

In Sub-Saharan agroforestry parklands, farmer management alter the composition (e.g., diversity) and structure (e.g., density) of the woody species communities through silviculture (e.g., tree pruning and thinning), tree planting, as well as assisted regeneration, and fire protection of trees. While the assisted regeneration and tree planting increase the storage of biomass carbon, the harvest of woody biomass through pruning and thinning (thereafter referred to as tree harvesting) can reduce AGC stock, especially when performed at high intensity^7,46^. Besides, parkland management practices targeting the tree component, crop fertilization and livestock grazing can influence soil physical and chemical properties through nutrient addition or otherwise nutrient mining and soil compaction.

**Figure 2:**
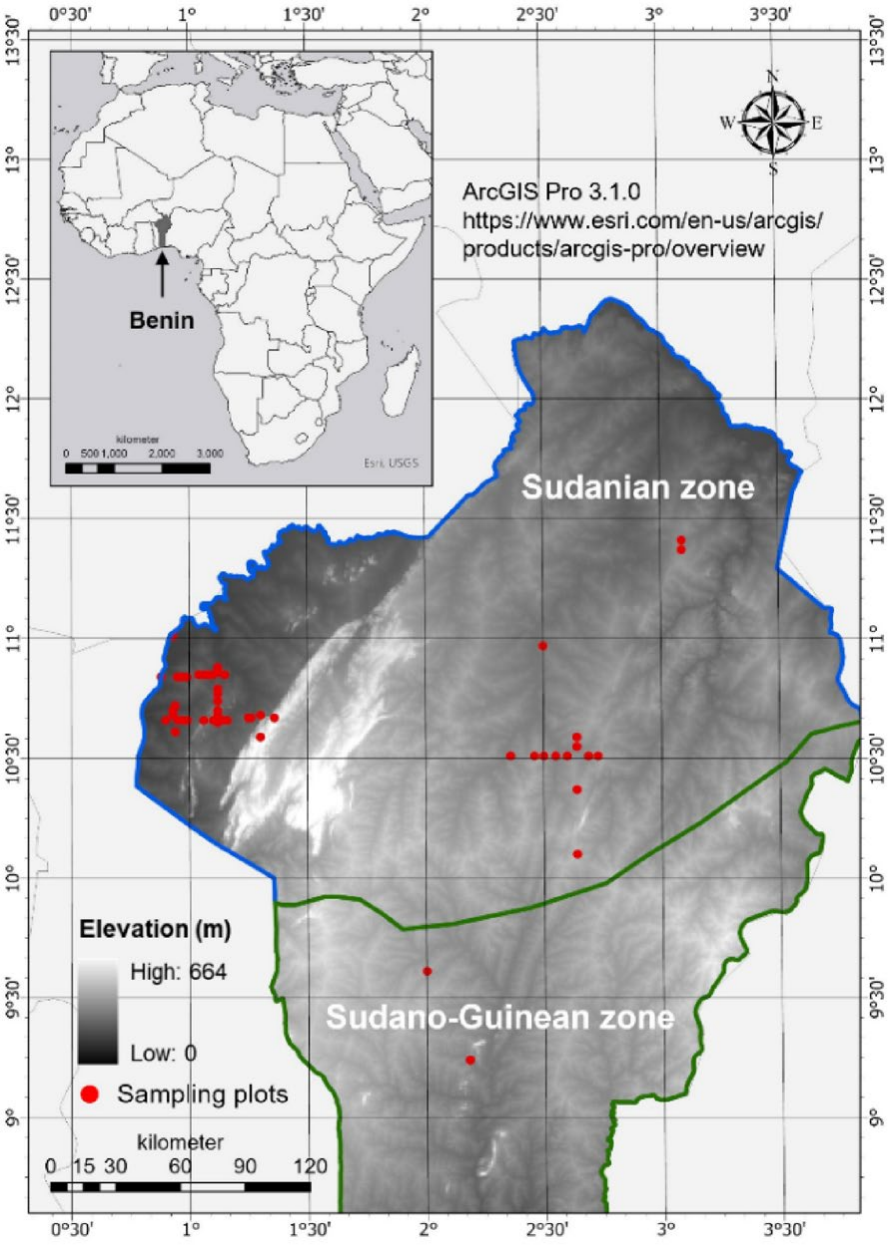
Map of the study area showing the distribution of sampling plots across the Sudanian and Sudano-Guinean climatic zones of Benin. Additional information on the layout of the sampling points is provided in Fig. S2. The map was elaborated by the authors in 2023.

In this study, we examined the relative importance of human management (i.e., tree harvesting), abiotic (i.e., climate, topography, and soil) and biotic (i.e., diversity attributes of woody species) factors for AGC storage in agroforestry parklands in the drylands of northern and upper-central Benin (Fig. 2 and Fig. S1). The study area encompasses semi-arid and dry sub-humid regions, where parklands are particularly prominent^8^. We employed linear mixed-effects models and structural equation modeling (SEM), as a powerful integrative framework^47^ to address three main questions (Fig. 1):

1. What are the direct effects of the multiple drivers of AGC stocks and what is their relative importance? Given that parklands reportedly had lower woody species diversity than the adjacent natural forests^10,48^, we hypothesized that the diversity attributes of woody species have positive and stronger effects than the other drivers because any additional species will substantially contribute to AGC storage due to low species redundancy^27^.
2. What are the indirect effects of abiotic factors on AGC stocks, as mediated by the diversity attributes of woody species? We hypothesized that increasing resource availability increases AGC stocks through its positive effect on the diversity attributes of woody species.
3. What are the effects of human management on AGC stocks, as mediated by both the abiotic factors and diversity attributes of woody species? We hypothesized that the harvest of woody biomass decreases soil nutrient content, diversity attributes of woody species, and AGC stocks.

## Materials and methods

### Study area and layout of sampling plots

We used data from 51 circular plots of 0.1 ha^49^, delineated in the Sudanian and Sudano-Guinean zones of Benin (Fig. 2). The study area belongs to the Sudano-Sahelian zone, which supports a wide spectrum of abiotic and biotic heterogeneity and human impact histories^50,51^. The inventory plots were systematically installed on four concentric circles of 5, 10, 15 and 20 km of radius in the north–south and west–east directions, following the land degradation surveillance framework^49^. Each set of four concentric circles was located within a grid of 50 km × 50 km; the grids were distributed across the study area (Fig. S2). The vegetation in the study area is dominated by dry and gallery forests, tree and shrub savannas and agroforestry parklands^52^. In this study, only the parklands plots are reported (Fig. S1). These plots spread over four municipalities (i.e., Atacora, Borgou, Alibori and Donga) of the country and span across the semi-arid and dry sub-humid climate zones, with an aridity index value between 0.4 and 0.6 (Table S1; Fig. 2). The mean annual temperature (MAT) ranged from 26.3 to 28.6 °C and the mean annual precipitation (MAP) from 917 to 1127 mm. The elevation ranged from 146 to 483 m a.s.l. (Table S1). The soil types included tropical ferruginous and hydromorphic soils^53^.

### Plot inventory data

Field inventories took place between 2019 and 2021. Within each sampling plot, all live individual trees, and shrubs with a diameter at breast height (1.3 m height—DBH, cm) ≥ 5 cm were identified to the species level, counted, and measured for their DBH, height (m) and crown diameter (m). Heavily pollarded live trees were excluded from these measurements. For the trees bifurcating below 1.30 m, the DBH of all ramifications was measured and the quadratic mean diameter was calculated as the square root of the sum of squares of DBH of individual stems^54^. The crown diameter was the average of the distance of two vertical north–south (d_1_, m) and east–west (d_2_, m) projections of the crown on the ground surface^55^. Latin names of the enumerated tree species were identified following the flora of Benin^56^. Nine to 15 leaves were collected from two to three trees of the dominant species (representing > 75% of the total basal area at plot-level) per plot. The leaves were sampled from the upper, middle, and lower parts of the tree canopy to account for heterogeneity in light capture and bulked per tree. The fresh and dry weights of three to five selected leaves per tree were measured and used to determine the leaf dry matter content (LDMC, mg g^−1^)^57^. The remaining leaves were bulked, milled, and analyzed for the leaf total carbon (LC%) and nitrogen (LN%) content.

Soil samples were collected from the center of four subplots of 0.01 ha installed within the main plot^49^ at three depths: 0–20 cm, 20–40 cm and 40–60 cm. These samples were mixed according to the sampling depth to form composite samples, which were analyzed for their total carbon (C%) and nitrogen content (N%). The soil C% ranged from 0.22 to 2.01% and the soil N% ranged from 0.03 to 0.50% (Table S1). The C% and N% of the leaf and soil samples were analyzed with a vario MACRO cube CHNOS elemental analyzer (Elementar Analysensysteme GmbH, Germany).

### Vegetation characteristics and management of parklands

We identified 52 woody species, belonging to 38 genera and 19 families across the 51 plots in the parklands (Tables 1 and Table S2). The woody species included trees, shrubs, and palm species (thereafter referred to as trees), which were either deciduous, semi-deciduous or evergreen. The dominant species, judged by their importance value index (IVI) ranging 24–62, included *Vitellaria paradoxa* C.F.Gaertn., followed by *Parkia biglobosa* (Jacq.) R.Br. ex G.Don and *Adansonia digitata L.* (Tables 1 and Table S2). The average density of woody species in the parklands was 73 trees ha^−1^ (Table S1). Maize (*Zea mays* L.), cotton (*Gossypium hirsutum* L.), sorghum (*Sorghum bicolor* L. Moench) and yam (*Dioscorea* spp L.) were the common annual crops grown in the parklands. Pruning and thinning (thereafter harvesting) of the woody species were the major management practices applied to trees and shrubs in the parklands. Pruning was typically performed on young trees by removing the lower scaffold branches and twigs. The main purposes of tree harvesting included firewood provision, charcoal production, and shade reduction for the associated crops. Farmers applied organic and mineral fertilizers to the annual crops, which might indirectly benefit the woody species. To protect the woody species in the parklands against fires, farmers practice controlled burning at the start of the dry season^7^. Small ruminants and cattle are often allowed to graze in the parklands for a limited time during the dry season^10^.

**Table 1:** Characteristics of the ten most important woody species in the parklands, as judged by their Importance Value Index (IVI).

Species name	Life form^a^	Leaf habit^a^	IVI^b^	Density^c^	DBH (mean ± SD)	Height (mean ± SD)
*Vitellaria paradoxa* C.F.Gaertn	Tree	Deciduous	61.69	90	62.97 ± 44.32	8.77 ± 2.74
*Parkia biglobosa* (Jacq.) R.Br. ex G.Don	Tree	Deciduous	43.24	42	78.20 ± 70.13	11.11 ± 4.93
*Adansonia digitata* L	Tree	Deciduous	23.96	21	110.95 ± 61.55	13.36 ± 4.79
*Azadirachta indica* A. Juss	Tree	Evergreen	12.74	26	18.93 ± 12.93	7.39 ± 2.44
*Lannea microcarpa* Engl. & K. Krause	Tree	Deciduous	11.96	17	43.49 ± 20.38	7.68 ± 2.01
*Daniella oliveri* (Rolfe) Hutch. & Dalziel	Tree	Deciduous	11.56	5	199.80 ± 65.28	16.84 ± 1.76
*Borassus aethiopum* Mart	Palm	Evergreen	10.56	17	56.61 ± 29.16	10.41 ± 3.27
*Bombax costatum* Pellegr. & Vuillet	Tree	Deciduous	7.98	9	64.44 ± 63.45	8.77 ± 5.82
*Terminalia microcarpa* Decne	Tree	Semi-deciduous	7.91	9	84.65 ± 44.64	10.63 ± 5.26

A full list of all the 52 species is presented in Table S2.

*SD* standard deviation.

^a^The life form and leaf habit of the species were extracted from the “Useful Tropical Plants” database^71^.

^b^The IVI was computed as the sum of the relative frequency, relative density and relative dominance (Supplementary Information, Methods) following Kifle et al.^103^.

^c^Density represents the total number of individuals of a species across the 51 sampling plots.

### Aboveground carbon stocks

We used the pan-tropical allometric equation developed by Chave et al.^58^ for the world’s tropical forests (Eq. 1) to estimate the aboveground biomass (AGB, kg) of individual trees:

1 $$AGB=0.0673 \times {\left(\uprho \times {DBH}^{2} \times height\right)}^{0.0976}$$

where ρ is the species wood density in g∙cm^−3^, DBH is the diameter at breast height and height is the total tree height. The generic Chave et al.^58^ equation has been widely applied to estimate the AGB of individual trees across vegetation types in the tropics^40,42,59,60^, including natural and managed vegetation types in West Africa^40,60,61^. It has been suggested that AGB quantification following Chave et al.^58^ produced acceptable estimates of stand-level biomass-related aspects^40,60^, thereby confirming the utility and suitability of this equation for our quantification of plot-level AGB^40,60^ in absence of allometric equations specific to the study region or woody species encountered. The ρ value for each tree species was retrieved from local studies in West Africa^62,63^ and the Global Wood Density Database^64^. We then calculated the AGC stock of individual trees by multiplying the AGB by the conversion factor of 0.456^65^. The plot-level AGC stock was calculated by summing the AGC stock of all individual trees within a plot and estimated in Mg ha^−1^.

### Predictor variables

We used 32 potential variables that described abiotic, biotic, and human management factors to characterize each sampling plot (Table S3). The biotic variables included metrics of taxonomic, functional, and structural diversity. We quantified the taxonomic diversity using the Shannon species diversity (H_SR_; Supplementary Methods) because it accounts for species richness and evenness and is considered to be an appropriate measure of species diversity in species-poor ecosystems^36^ such as the parklands in our study area (80% of the 51 plots consisted of less than 5 tree species). The functional diversity was quantified based on four widely-used metrics: functional richness (F_rich_), functional evenness (F_eve_), functional divergence (F_div_) and functional dispersion (F_dis_)^66,67^. The community-weighted mean (CWM) of functional traits was used as a measure of functional identity (Supplementary Methods). The H_SR_ was estimated for each plot using the “ChaoShannon” from the R package “iNEXT” to account for rarefied species and variation in sampling effort among plots^68^. The function returns the estimated asymptote for H_SR_ based on rarefaction and extrapolation curves^68,69^.

The functional diversity and identity metrics were computed at the plot level based on the relative basal area of the species and 10 functional traits: ρ, maximum tree height (MAXH, m), specific leaf area (SLA, mm^2^ mg^−1^), leaf area (LA, cm^2^), LC%, LN%, LDMC, leaf habit (LH; deciduous or evergreen), nitrogen fixation (NF; nitrogen fixer or non-fixer) and maximum crown area (MAXCA, m^2^; Supplementary Methods). Apart from the values of SLA and LA obtained for each species from the TRY database^70^, the values of MAXH, LC%, LN%, LDMC and MAXCA were obtained from field measurements. The data on the LH and NF (Table S2) were retrieved from the Useful Tropical Plants Database^71^. The selected traits are related to leaf economics and tree size, which underpin plant life-history strategies^72,73^. We used genera or family values for the species with no available trait data. When the genera or family value was unavailable for a species, we used the imputation method suggested by Josse and Husson^74^ to fill the missing data^75^. In the end, 100% of the species had values of ρ, MAXH, LH, NF and MAXCA, while values of SLA, LA, LC%, LN% and LDMC were imputed for 25%, 23%, 17%, 6% and 17% of the species, respectively. The functional diversity and identity attributes were computed using the R package “FD”^76^.

The Shannon structural diversity index for DBH (H_DBH_) and height (H_height_) and the coefficient of variation of DBH (CV_DBH_), height (CV_height_) and CA (CV_CA_) were used to characterize the structural diversity (Supplementary Methods)^33,34^. These metrics are related to the vertical and horizontal architectural structure of trees within a stand. The H_DBH_ and H_height_ measure the richness (number of classes) and evenness of stand structural diversity by giving equal weight to horizontal and vertical diversity^34^ (see the formulas in Supplementary Methods). The computation of H_DBH_ and H_height_ requires the grouping of the data into arbitrary DBH and height classes to calculate proportions. Both indices are insensitive to tree size, as they are weighted by the proportion of basal area^34^. Here, we calculated the H_DBH_ and H_height_ for three discrete DBH (5, 10 and 15 cm) and height (4, 8 and 12 m) classes and selected the most representative class for further analyses (see the following section for the variable selection procedure). Further information on the computation of the species diversity attributes is provided in the Supplementary Information, Methods.

Potential abiotic factors were collated for each plot based on the geographic coordinates of the plot center. The abiotic factors included climate, topography, and soil physical and chemical properties. We used climatic moisture index (CMI) to represent climate-related water availability following previous studies^77–79^. The CMI was computed as the difference between the MAP and mean annual potential evapotranspiration (PET) and thus indicates potential aridity level. Data on MAP and PET were extracted from the WorldClim2 database at a resolution of 30 arcseconds (~ 1 km) for the period 1971–2000^80^. We multiplied the CMI values by –1 since CMI is negative by definition^16^. Thus, sites with higher CMI values are seasonally drier. We used the elevation data recorded in the field to characterize topography. In addition to the soil C% and N% measured in the field-collected samples, we downloaded data on clay content (%), silt content (%), sand content (%) and cation exchange capacity (CEC, cmol kg^−1^) for four standard soil depth intervals: 0‒5, 5‒15, 15‒30, and 30‒60 cm. The soil data were obtained from the Africa Soil Profiles Database at a resolution of 1 km^81^. The weighted mean values of the soil variables for a soil depth of 60 cm were used for the analyses.

We used the number of harvested trees and shrubs (NHT) per plot as an indicator of human management of woody biomass and hence AGC stock because of the local farmers’ practice to harvest tree and shrub biomass as firewood, to produce charcoal and increase space for adjacent crops. We consider a tree or shrub as “harvested’ when it was felled or pruned. Here we consider the NHT as a proxy for assessing the impact of human management on the parkland woody species in the absence of experimentally collected data on harvest intensity (e.g., proportion of crown or biomass harvested). The average NHT was 13 trees per plot (Table S1).

### Statistical analysis

Prior to the analyses, we used the Shapiro–Wilk test to assess the normality of all potential variables and log- or square root-transformed those variables which did not meet the normality assumption. Then, we standardized all variables using the “scale” function in R to allow for reasonable coefficient comparisons. Given that the soil properties, functional diversity, functional identity, and structural diversity were represented by several variables, we performed multimodel inferences and model selection to choose the most important variables for each of these groups. This was done to minimize complexity and avoid multicollinearity and model overfitting in the subsequent analyses. We constructed candidate linear mixed-effects models (LMMs) based on all possible combinations of co-variables and averaged them to obtain the most parsimonious model using the “dredge” and “model.avg” functions of the R package “MuMIn”^82^, respectively. The LMMs included AGC stock as the dependent variable. We used region identity as a random factor in the LMMs to account for unknown regional differences. Only the variables with low variance inflation values (VIF < 5) were included in the LMMs (Table S3). Subsequently, we calculated the relative variable importance based on the averaged model and chose the variable with the highest importance value as the most representative variable. As a result, the C%, community-weighted mean of MAXH (CWM_MAXH_) and H_DBH_ for the 15 cm DBH class (thereafter referred to as H_DBH_) were chosen as the most representative predictors of soil properties, functional identity, and structural diversity, respectively (Table S3).

**Figure 3:**
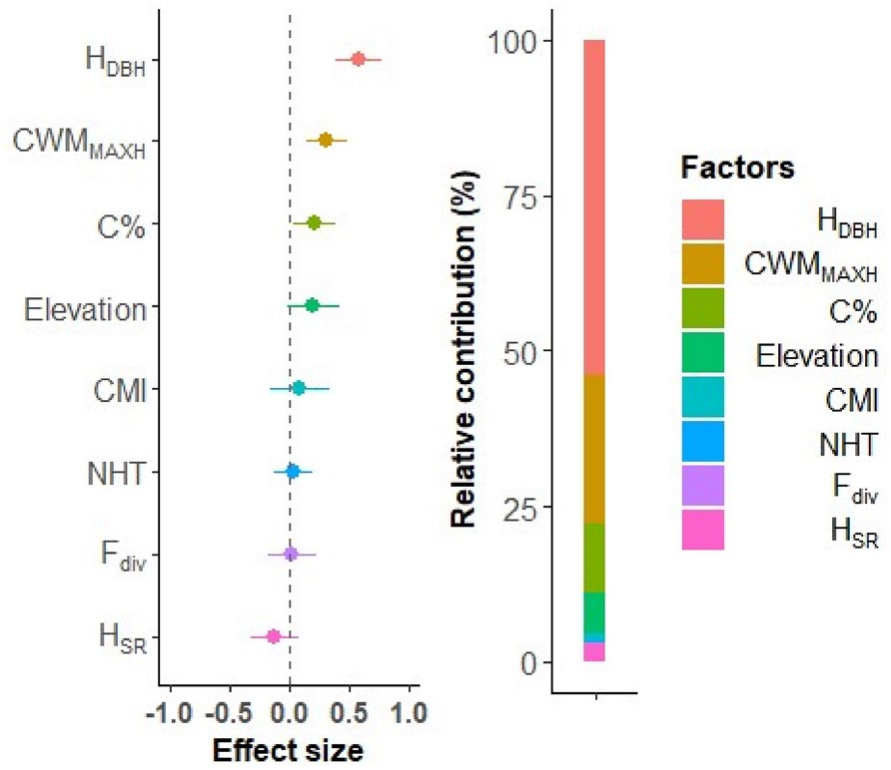
Effect size and relative contribution of human management (NHT), abiotic (CMI, Elevation and C%) and biotic (H_SR_, F_div_, CWM_MAXH_ and H_DBH_) factors on aboveground carbon (AGC) stocks. The effect size (i.e., dots) represents the standardized coefficients from the multiple linear mixed-effects models (LMMs), which are shown along with their 95% credible intervals (horizontal lines). The 95% credible interval that does not cross the vertical zero line indicates a significant effect. The standardized effect sizes are ordered from the highest positive effect to the lowest negative effect. The pseudo R^2^, representing the proportion of variance explained by both fixed and random effects, was 78%. The relative contribution represents the contribution of a variable to the explained variance, which was quantified by the partial pseudo-R^2^. A summary of the LMM output is provided in Table S4. *NHT* number of harvested trees, *CMI* climatic moisture index (multiplied by – 1), *C%* soil total organic carbon content, *H*_*SR*_ Shannon species diversity, *F*_*div*_ functional divergence, *CWM*_*MAXH*_ community-weighted mean of maximum height, *H*_*DBH*_ Shannon structural diversity index for 15 cm DBH class.

We modelled the direct effect of the selected human management, abiotic and biotic variables on AGC stocks and their relative importance using multiple LMMs, with region identity included as a random factor. Before running the regression analyses, we checked whether the selected predictor variables were highly correlated (i.e., r >|0.7|) by calculating the correlation matrix between them (Fig. S3). As a result, we found that the variables were not highly correlated (Fig. S3). The relative importance of each covariable was quantified by its relative contribution to the explained variance, expressed as the partial pseudo coefficient of determination (R^2^). The partial pseudo-R^2^ represents the variance explained by each covariate while accounting for the effects of other covariate present in the model. We used the “r2glmm” R package^83^. The relative contribution of the variables was further grouped according to the three main categories of predictors (Table S4). We used the Shapiro–Wilk test to assess the normality of the model’s residuals. The VIF values of the selected variables were also examined. We used the Moran’s I test to examine the effect of spatial autocorrelation in the residuals of the LMM. The Moran’s I test, conducted using the R package “spdep”^84,85^, showed that there was no strong influence of spatial autocorrelation on the coefficient estimates (Table S4). We graphically presented the standardized coefficients or effect size (β) from the multiple LMMs along with the relative variable contribution (Fig. 3).

To explicitly evaluate how pathways between multiple predictors regulate AGC stocks, we used piecewise SEM (pSEM). We constructed the conceptual pSEM based on demonstrated multivariate interrelationships between environmental conditions, species diversity and AGC stocks in tropical forests^15,60,77,79^, with the addition of potential effect of human management (Fig. 1). A key consideration in our decision to use pSEM is that it expands on the traditional SEM and enables the inclusion of both simple linear and mixed-effects regressions in the modeling framework, which is challenging in the traditional SEM^86^. The component regression models can be evaluated separately and then combined later to generate inferences about the whole SEM. We fitted the component models of the pSEM as LMMs, with region identity included as a random variable. Only the selected human management, abiotic, and biotic variables were included in the pSEM. We evaluated the model fit based on the Fisher's C statistic. The pSEM was considered to have an adequate fit to the data when the model had a Fisher's C statistic with p > 0.05^87^. We performed a confirmatory path analysis based on the directional separation test in pSEMs^87^ to evaluate whether missing significant paths between variables should be added to the model either as direct paths or correlated errors. We calculated the conditional R^2^ (R^2^_c_) and marginal R^2^ (R^2^_m_) for each of the dependent variables. The R^2^_c_ reflects the variance explained by both fixed and random variables, whereas the marginal R^2^_m_ reflects the variance explained by fixed factors only^88^. As pSEM does not compute indirect effects, we manually calculated them for each response variable by multiplying the values of all direct paths (i.e., direct effects) linking two variables through a mediator variable. The pSEM was fitted using the R package “piecewiseSEM”^86^. We complemented the results of the SEM by running additional LMMs assessing the influence of human management and abiotic variables on each biotic variable. Finally, we assessed the bivariate relationships of the causal pathways hypothesized in the SEMs using simple linear regression analysis (Figs. S4, S5). All statistical analyses were conducted in R version 4.0.2^89^.

## Results

### Direct effects of multiple predictors on AGC stock

The fixed factors of the multiple LMM including human management, abiotic and biotic factors explained 61% of the total variance in AGC stock (Fig. 3). The biotic factors contributed the most to the explained variance in AGC stock, with a relative contribution of 81% (Table S4). Subsequently, the H_DBH_ was the most important driver of AGC stock, with the largest effect size (β = 0.59) and relative contribution (54%) to the overall explained variance (Fig. 3). This was followed by the CWM_MAXH_, which had the second largest effect size (β = 0.32) and relative contribution (24%). AGC stock increased with increasing H_DBH_ and CWM_MAXH_. The H_SR_ and F_div_ did not have significant effects on AGC stocks. Among the abiotic factors, C% was the only significant driver and had a positive effect (β = 0.21) on AGC stocks. The CMI, elevation and NHT did not have any substantial effects on AGC stocks (Fig. 3, Table S4).

### Interacting effects of multiple predictors on AGC stocks

**Figure 4:**
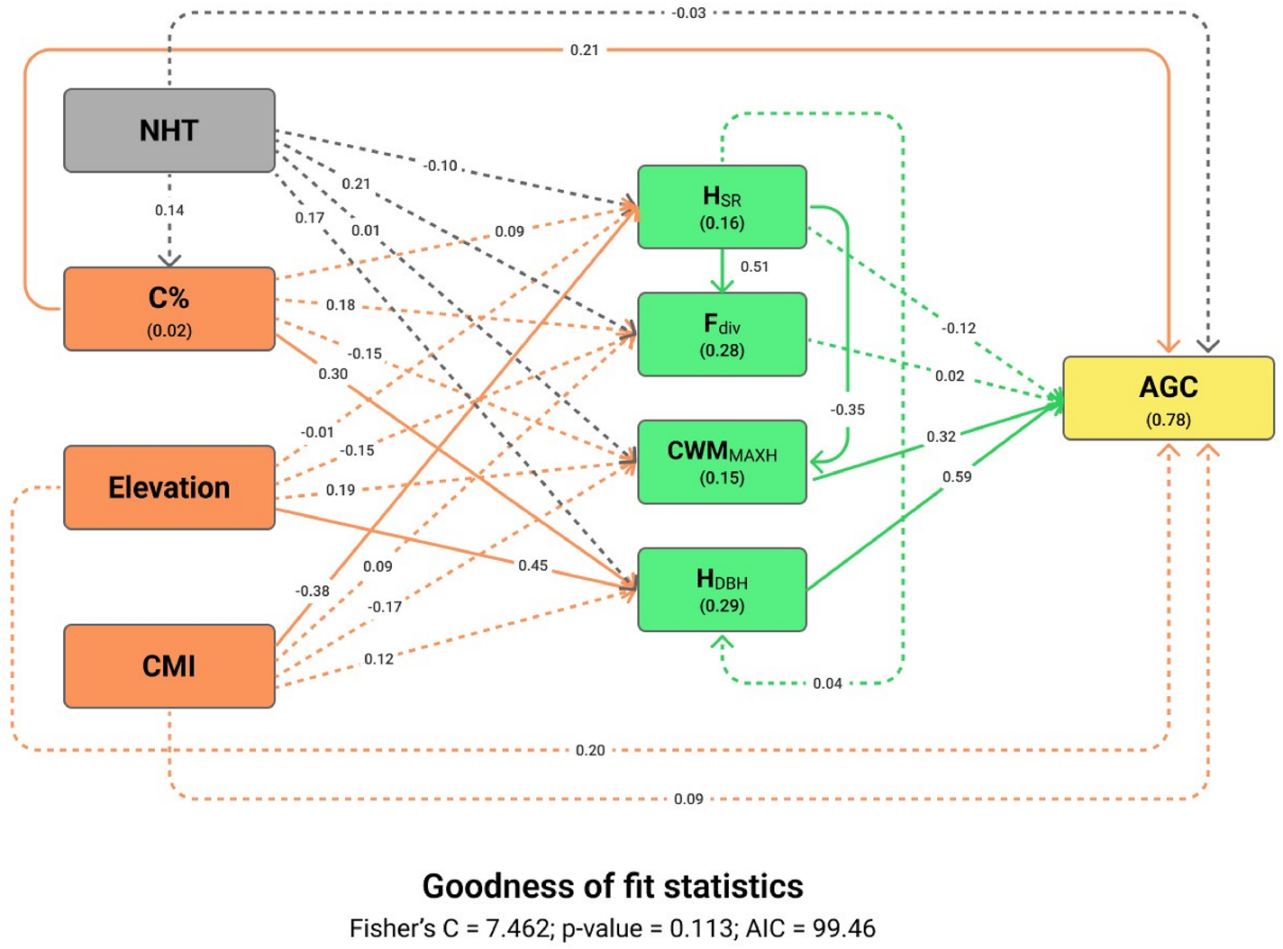
Output of the piecewise structural equation model (pSEM) linking human management (NHT), abiotic (CMI, Elevation and C%) and biotic (H_SR_, F_div_, CWM_MAXH_ and H_DBH_) factors to aboveground carbon (AGC) stocks. The values without brackets on the graphs are the standardized regression coefficients and their significance is shown in Table S5. The values within brackets are the conditional coefficients of determination (R^2^_c_), shown for the dependent variables. Single-headed arrows indicate the causal paths. Solid arrows represent significant paths (α < 0.05). Dashed arrows represent non-significant paths (α > 0.05). AIC is the Akaike information criterion. Other acronyms are described in Fig. 3.

**Figure 5:**
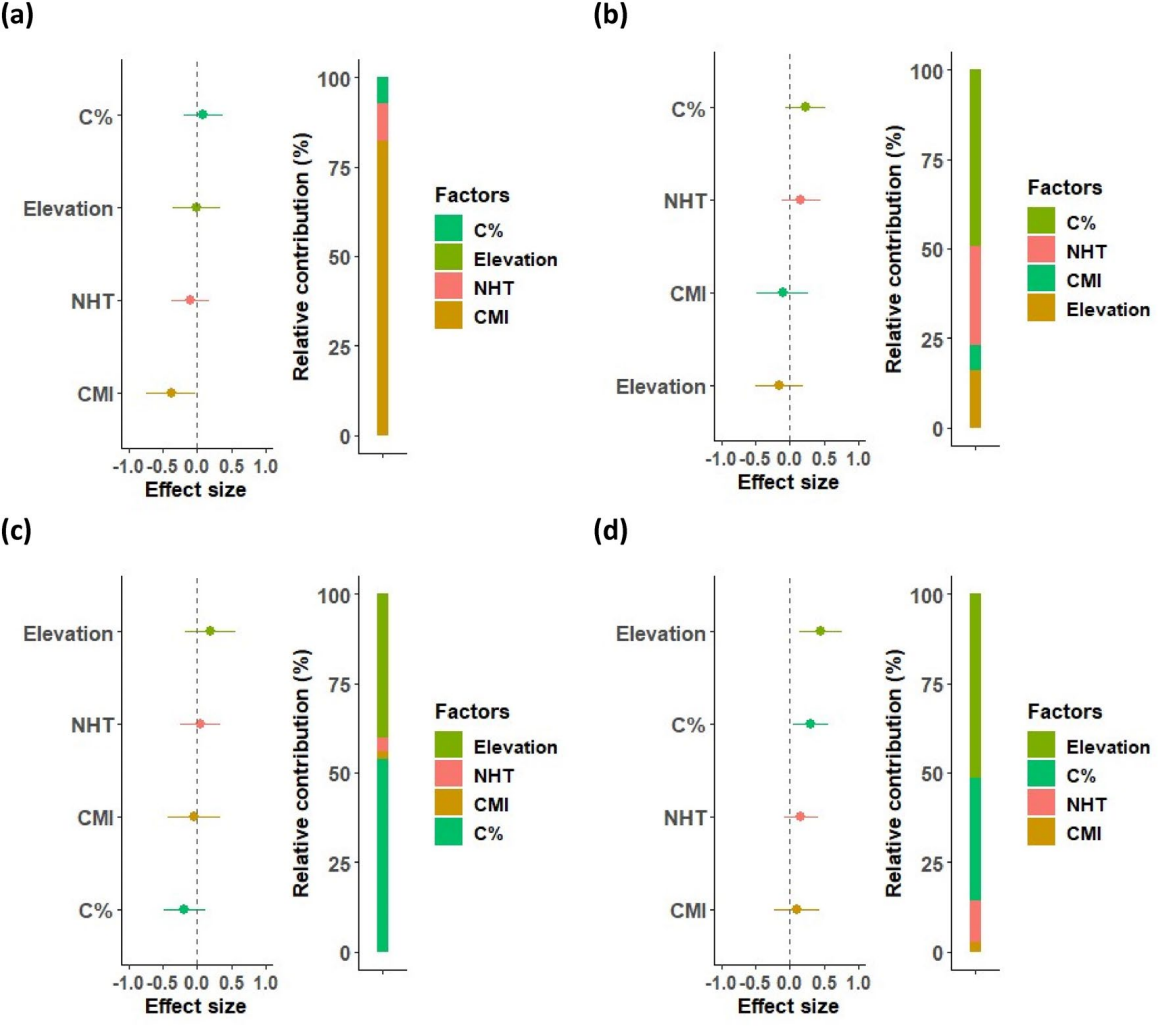
Effect size and relative contribution of human management (NHT) and abiotic (CMI, Elevation and C%) on H_SR_ ((**a**); pseudo R^2^ = 16%), F_div_ ((**b**); pseudo R^2^ = 8%), CWM_MAXH_ ((**c**); pseudo R^2^ = 6%) and H_DBH_ ((**d**); pseudo R^2^ = 31%). The effect size (i.e., the dots) represents the standardized coefficients from the multiple linear mixed-effects models (LMMs), which are shown along with their 95% credible intervals (horizontal lines). The 95% credible interval that does not cross the vertical zero line indicates a significant effect. The relative contribution represents the contribution to the explained variance, which was quantified by the partial pseudo-R^2^. Acronyms are described in Fig. 3.

The outputs of the pSEM revealed the indirect effects of abiotic and biotic factors on AGC stocks, which were mediated by H_DBH_, CWM_MAXH_ and H_SR_ (Fig. 4; Table S5). Although elevation, CMI and H_SR_ had no significant direct effects on AGC stocks (Fig. 3), they indirectly affected AGC stocks. Elevation had a positive indirect effect via H_DBH_ (β = 0.26). H_SR_ indirectly decreased AGC stocks through its negative effect on CWM_MAXH_ (β = −0.11; Fig. 4). CMI was marginally and positively linked to AGC stocks (β = 0.04), as it negatively influenced H_SR_, which in turn, had a negative indirect effect on AGC stocks. In addition to its positive direct effect on AGC stock, C% had an indirect positive effect via H_DBH_ (β = 0.18). The AGC stocks were not indirectly affected by NHT (Fig. 4; Table S5). The results of the additional multiple LMMs testing the effects of the abiotic factors on the biotic factors and that of the bivariate analysis supported the causal relationships observed in the pSEM (Fig. 5, S4). Most notably, H_DBH_ strongly increased with increasing elevation, whereas H_SR_ decreased with increasing CMI (Fig. 5).

## Discussion

Given that the tropical forest cover continues to decline^90^ while tree cover on agricultural land has increased^4,91^ offering key nature-based solutions for climate change mitigation and adaptation, understanding the drivers of carbon storage in agroforestry parklands is increasingly needed. To the best of our knowledge, this study is one of the few that explored the multivariate relationships between AGC stocks and several drivers including human management, environmental conditions, and diversity attributes in agroforestry parklands. It should be noted that although our study establishes empirical trends, our results are largely based on correlative trends derived from the observational data. Our estimate of AGB presents some limitations in that we could not particularize the effect of pruning due to the lack of species-specific allometric equations for trees which undergo partial biomass removals.

We expected a stronger relationship of the diversity attributes with AGC stocks in the parklands given the low species richness (range 2 to 9), since the largest changes in carbon storage may occur at low levels of taxonomic, functional and structural diversity where low species redundancy favors the substantial contribution of each additional species (including its functional traits) to ecosystem functions^27,92^. Accordingly, we found support for the first hypothesis in that diversity metrics (H_DBH_ and CWM_MAXH_) were more strongly related to AGC stock estimates than human management (i.e., tree pruning and harvesting) and abiotic factors.

In particular, the major predictor of the variation in AGC stocks in the parklands was the tree diameter diversity, as evidenced by its strongest individual relationship with AGC stock and its weak correlation with H_SR_ and CWM_MAXH_ (Fig. S3). Our result is in line with findings of previous studies across forests and agroforestry systems in tropical and temperate areas, where H_DBH_ or DBH variation was the strongest driver of AGB and AGC as compared to diversity in species and functional traits^15,18,19,93,94^. The strong role of H_DBH_ in shaping AGC stocks extends the biological importance of structural diversity as a major driver of carbon storage to dryland agroforestry parklands. This finding suggests that the parkland plots that are more structurally complex favored a greater spatial niche differentiation between co-occurring woody species of diverse DBH sizes, which optimizes resource use efficiency, thereby increasing AGC stocks in these plots, in line with the predictions of niche complementarity^24^. In forests, greater H_DBH_ implies a complex horizontal structure which enhances canopy packing and aboveground light capture within a stand^19,95^. However, the studied parklands were characterized by an open canopy (Fig. S1) and a tree density (average of 73 trees ha^−1^; Table S1) lower than that of the forests (average of 434 tree ha^-1^, Noulèkoun et al. unpublished) in the same study area, suggesting that the growth-related impact of light availability may be more pronounced in plots with higher tree density. In this line, our results showed that H_DBH_ increased with increasing tree density, indicating that structurally more diverse stands contained a higher number of trees, which increased the AGC (Fig. S5). The positive effect of H_DBH_ on AGC stocks could also be that stands with higher H_DBH_ contained more of large trees, which stored more AGC^36,60^. Moreover, unlike studies showing that structural diversity mediates the effect of taxonomic diversity on biomass or carbon stocks in forests since higher number of species may result in greater diversity of tree morphologies^15,36^, this study demonstrated that H_SR_ does not influence H_DBH_ in the parklands_._ This finding corroborates the idea that species-poor stands can also be structurally diverse through horizontal differentiation of a small number of species^33^. Overall, in the parklands, structural diversity is more likely driven by tree density rather than by species diversity. This is evidenced both by the strong and positive association between H_DBH_ and tree density and by lack of significant effect of H_SR_ on H_DBH_ (Figs. S4 and S5).

In contrast to the previous findings reporting a positive direct effect of taxonomic diversity on biomass and carbon storage in tropical forests^15,27,60^ and agroforestry systems^17,18^, we found that H_SR_ did not directly influence AGC stocks in the parklands. A previous study that used H_SR_ to quantify species diversity in homegardens in the dry zone of Sri Lanka also reported a neutral direct effect of H_SR_ on AGB^96^. Instead, H_SR_ was negatively related to CWM_MAXH_, which in turn, had a positive effect on AGC stocks. In the drylands of West Africa, selective removal of seedlings and saplings in favor of crop cultivation on same land has halted natural regeneration of trees with a subsequent decline of species pool and ageing of the parkland trees, prompting a higher prevalence of older, larger-diameter and taller trees^7,10,48,48,97^. The latter phenomenon was also evidenced in our study (Table 1 and Table S2). Therefore, on the one hand, the lack of significant direct effect of H_SR_ on AGC stocks and its negative relationship with CWM_MAXH_ may thus be attributed to the effect of human-driven filtering of parkland species, resulting from the exclusion of regenerating woody species and logging preference for woody species of low socio-economic value to the farmers^10^. On the other hand, the positive effect of CWM_MAXH_ on AGC stock reflects a dominance effect and indicates that carbon storage in the parklands is also shaped by the high contribution of the tall-stature species, in support to the mass-ratio hypothesis. Therefore, our results suggest that the effect of H_SR_ on AGC stocks is also likely driven by the presence of highly productive species that dominate ecological processes by extirpating non-productive or weak species from assemblages through interspecific interactions, such as competition^96,98^. The findings further highlight the potential role of human-driven filtering of species in regulating the effect of biodiversity on carbon storage in human-modified landscapes.

We expected that abiotic factors and human management would modulate the relationships between biotic factors and AGC stock (H2 and H3). In line with this, we found substantial direct and indirect (via H_DBH_) effects of C% and elevation on AGC stocks, whereas CMI indirectly increased AGC stocks through its effects on species diversity (Fig. 4). We also found that higher elevations were associated with a higher water availability, and a higher tree density (Figs. S3–S5). Thus, the increase in H_DBH_ with increasing elevation suggests that the high-water availability at higher elevations promoted a greater stem density which increased the likelihood of co-occurrence of woody species having a variety of size through niche complementary, while simultaneously providing favorable environmental conditions for tree growth. These favorable species-environment interactions resulted in the storage of larger amount of AGC stocks at high elevations. Our results are reminiscent of the findings of Wen et al.^93^ and Chun et al.^41^ demonstrating the beneficial role of high resource availability at high elevations on species diversity and biomass carbon in forests. The positive direct and indirect effects of C% on AGC stocks also indicate the importance of nutrient availability in modulating not only AGC storage, but also the diversity and adaptation of species with different diameter size to local soil conditions in the parklands, as previously reported^77,93^. The occurrence of high species diversity in more moist sites further supports the role of environmental favorability in promoting species diversity^39^ and AGC, even though the effect of species diversity did not directly translate to a greater carbon storage, but rather indirectly through the dominance of tall-stature trees. Collectively, our findings revealed that abiotic drivers influenced AGC storage through both biotic mechanisms and water and carbon availability in the agroforestry parklands, supporting our second hypothesis (H2).

We found neither direct nor indirect significant associations between human management (here tree harvest expressed as the NHT) and soil C%, species diversity attributes, and AGC stocks in the parklands. This observation is consistent with the findings of previous studies where biomass removal for subsistence use (e.g., gathering fuelwood and cutting small poles) had no noticeable effect on biomass and carbon stocks in tropical forests^45,99^. Four possible reasons could explain the lack of significant effects of NHT on AGC stocks. First, the current selective maintenance and tree harvest practices did not significantly affect vegetation characteristics and decrease AGB at the plot level^45^. Second, the expected negative effect of tree harvesting on C% might have been counterbalanced by the supply of mineral and organic fertilization of crops in the parklands. Third, other management factors, such as controlled fires (by burning of crop residues) and livestock grazing, reported to impede tree regeneration^100,101^, could have overlaid the NHT influence on species diversity and AGC stocks. However, such effects were not evaluated in this study because of the insufficient fire occurrence data (only 11% of the plots had fire frequency records according to MODIS Burned Area Product-MCD64A1^102^) and because grazing occurs for a limited time and in few (< 30% of the sampling plots) parklands, as also observed by N’Woueni and Gaoue (2021) in agroforestry systems in north-western Benin. Lastly, other indicators of human management such as those that quantify the amount of biomass loss due to tree harvesting may have had a stronger effect on AGC than the NHT. However, the NHT was preferred in this study because it allowed to account for tree pruning practice in the parklands. The use of an indicator of human management based on the biomass or basal area of stumps was however challenged by the difficulty of quantifying the proportion of biomass removed.

In this study, we provided a multivariate assessment of the major drivers of AGC stocks in agroforestry parklands in the dryland West Africa with implications for carbon storage and biodiversity conservation in parklands, which mostly harbored native species. First, the positive effects of H_DBH_ and CWM_MAXH_ on AGC stocks suggests that initiatives aimed at mitigating climate change through the promotion of agroforestry parklands would strongly benefit from maintaining high levels of structural diversity and functionally dominant species with acquisitive resource-use strategies^18,73,96^. However, dominance of large trees may lead to shading, thereby decreasing the yield of understory crops. Therefore, future investigations may determine the optimum levels of structural diversity and functionally dominant species to maintain shading level acceptable for the productivity of understory crops in the parklands. Second, the negligible influence of H_SR_ on AGC stocks reveals that conservation strategies favoring carbon sequestration may fail to simultaneously conserve biodiversity. Therefore, add-on strategies such as species enrichment planting and retention of juvenile trees would be needed to promote and protect biodiversity in the parklands. Last, the storage of AGC was strongly dependent on soil nutrient and water availability, suggesting that adequate management practices of neighboring crops from which parkland trees also benefit would be crucial in sustaining the AGC storage in the agroforestry parklands.

## Conclusions

The main findings of this study suggests three mechanistic underpinnings of AGC storage in agroforestry parklands, which support previous theoretical predictions: (1) tree size diversity (representing complementarity effect) and dominance of tall trees (representing mass ratio effect) determined high AGC stocks with a greater effect of the former; (2) species taxonomic diversity did not influence AGC stocks directly, but rather indirectly via the dominant species maintained by farmers; (3) high moisture and soil carbon availability and higher elevations promoted AGC stocks. We argue that the abiotic and biotic factors are important determinants of AGC stocks in the parklands. We expect that our findings advance the existing knowledge base on biodiversity-ecosystem functioning relationships by adding new insights on the complex interactions between human management, abiotic environment, biodiversity, and carbon storage in agroforestry parklands. Pending on availability of quantitative data on management practices in parklands, our methodology might be employed to test the effects of a broader set of human management drivers on AGC storage. Future experimental studies will also need to explore causal relationships between abiotic and biotic factors and AGC stocks.

### Supplementary Information

Supplementary Information.

Supplementary Figure S1.

Supplementary Figure S4.

Supplementary Figure S5.

## Data Availability

Data supporting the findings of this study and the R code used for data analysis will be made available on request.

## References

[1] Albrecht A, Kandji ST (2003). Carbon sequestration in tropical agroforestry systems. Agric. Ecosyst. Environ..

[2] Nair PKR (2011). Agroforestry systems and environmental quality: Introduction. J. Environ. Qual..

[3] Schroth G, McNeely JA (2011). Biodiversity conservation, ecosystem services and livelihoods in tropical landscapes: Towards a common agenda. Environ. Manag..

[4] Zomer R, Bossio D, Trabucco A, Van Noordwijk M, Xu J (2022). Global carbon sequestration potential of agroforestry and increased tree cover on agricultural land. Circ. Agric. Syst..

[5] Stavi I, Lal R (2013). Agroforestry and biochar to offset climate change: A review. Agron. Sustain. Dev..

[6] Lorenz K, Lal R (2014). Soil organic carbon sequestration in agroforestry systems. A review. Agron. Sustain. Dev..

[7] Boffa, J. M. *Agroforestry Parkland Systems in Sub-Saharan Africa* (1999).

[8] Nair, P. K. R. *An Introduction to Agroforestry*. (Kluwer Academic, 1993).

[9] Fifanou VG, Ousmane C, Gauthier B, Brice S (2011). Traditional agroforestry systems and biodiversity conservation in Benin (West Africa). Agrofor. Syst..

[10] N’Woueni DK, Gaoue OG (2021). Species ethnobotanical values rather than regional species pool determine plant diversity in agroforestry systems. Sci. Rep..

[11] Tadesse E, Abdulkedir A, Khamzina A, Son Y, Noulèkoun F (2019). Contrasting species diversity and values in home gardens and traditional parkland agroforestry systems in ethiopian sub-humid lowlands. Forests.

[12] Luedeling E, Neufeldt H (2012). Carbon sequestration potential of parkland agroforestry in the Sahel. Clim. Change.

[13] Nair PKR, Kumar BM, Nair VD (2009). Agroforestry as a strategy for carbon sequestration. J. Plant Nutr. Soil Sci..

[14] Tsedeke RE, Dawud SM, Tafere SM (2021). Assessment of carbon stock potential of parkland agroforestry practice: The case of Minjar Shenkora; North Shewa, Ethiopia. Environ. Syst. Res..

[15] Noulèkoun F (2021). Structural diversity consistently mediates species richness effects on aboveground carbon along altitudinal gradients in northern Ethiopian grazing exclosures. Sci. Total Environ..

[16] Pyles, M. V. *et al.* Human impacts as the main driver of tropical forest carbon. *Sci. Adv.* **8**, eabl7968 (2022). 10.1126/sciadv.abl7968 PMC9205592 35714191

[17] Ma Z, Chen HYH, Bork EW, Carlyle CN, Chang SX (2020). Carbon accumulation in agroforestry systems is affected by tree species diversity, age and regional climate: A global meta-analysis. Global Ecol. Biogeogr..

[18] Rahman MM, Kundu GK, Kabir ME, Ahmed H, Xu M (2021). Opposing ecological strategies together promote biomass carbon storage in homegardens agroforestry of southern Bangladesh. Forests.

[19] Aponte C (2020). Structural diversity underpins carbon storage in Australian temperate forests. Glob. Ecol. Biogeogr..

[20] Mensah, S., van der Plas, F. & Noulekoun, F. Do functional identity and divergence promote aboveground carbon differently in tropical semi-arid forests and savannas? *Ecosphere* (2021).

[21] Mensah S, Veldtman R, Assogbadjo AE, Kakaï RG, Seifert T (2016). Tree species diversity promotes aboveground carbon storage through functional diversity and functional dominance. Ecol. Evol..

[22] Zhang Y, Chen HYH, Reich PB (2012). Forest productivity increases with evenness, species richness and trait variation: A global meta-analysis. J. Ecol..

[23] Loreau M (2001). Biodiversity and ecosystem functioning: Current knowledge and future challenges. Science.

[24] Tilman D (1997). The influence of functional diversity and composition on ecosystem processes. Science.

[25] Grime JP (1998). Benefits of plant diversity to ecosystems: Immediate, filter and founder effects. J. Ecol..

[26] Chisholm RA (2013). Scale-dependent relationships between tree species richness and ecosystem function in forests. J. Ecol..

[27] Poorter L (2015). Diversity enhances carbon storage in tropical forests. Glob. Ecol. Biogeogr..

[28] Finegan B (2015). Does functional trait diversity predict above-ground biomass and productivity of tropical forests? Testing three alternative hypotheses. J. Ecol..

[29] Huang X, Su J, Li S, Liu W, Lang X (2019). Functional diversity drives ecosystem multifunctionality in a Pinus yunnanensis natural secondary forest. Sci. Rep..

[30] Wen Z (2019). Functional diversity overrides community-weighted mean traits in linking land-use intensity to hydrological ecosystem services. Sci. Total Environ..

[31] Fotis AT (2018). Above-ground biomass is driven by mass-ratio effects and stand structural attributes in a temperate deciduous forest. J. Ecol..

[32] Lin D (2016). Traits of dominant tree species predict local scale variation in forest aboveground and topsoil carbon stocks. Plant Soil.

[33] Brassard BW, Chen HYH, Wang JR, Duinker PN (2008). Effects of time since stand-replacing fire and overstory composition on live-tree structural diversity in the boreal forest of central Canada. Can. J. For. Res..

[34] Staudhammer CL, LeMay VM (2011). Introduction and evaluation of possible indices of stand structural diversity. Can. J. For. Res..

[35] Mensah S (2023). Structural and taxonomic diversity predict above-ground biomass better than functional measures of maximum height in mixed-species forests. Appl. Veg. Sci..

[36] Zhang Y, Chen HYH (2015). Individual size inequality links forest diversity and above-ground biomass. J. Ecol..

[37] Baumert S, Khamzina A, Vlek PLG (2016). Soil organic carbon sequestration in *Jatropha **curcas* systems in Burkina Faso. Land Degrad. Dev..

[38] Currie DJ (2004). Predictions and tests of climate-based hypotheses of broad-scale variation in taxonomic richness. Ecol. Lett..

[39] Harrison S, Spasojevic MJ, Li D (2020). Climate and plant community diversity in space and time. Proc. Natl. Acad. Sci. USA.

[40] Mensah S, Noulèkoun F, Dimobe K, Seifert T, GlèlèKakaï R (2023). Climate and soil effects on tree species diversity and aboveground carbon patterns in semi-arid tree savannas. Sci. Rep..

[41] Chun J-H, Ali A, Lee C-B (2020). Topography and forest diversity facets regulate overstory and understory aboveground biomass in a temperate forest of South Korea. Sci. Total Environ..

[42] Jucker T (2018). Topography shapes the structure, composition and function of tropical forest landscapes. Ecol. Lett..

[43] Scholten T (2017). On the combined effect of soil fertility and topography on tree growth in subtropical forest ecosystems—A study from SE China. J. Plant Ecol..

[44] de Avila AL (2018). Disturbance intensity is a stronger driver of biomass recovery than remaining tree-community attributes in a managed Amazonian forest. J. Appl. Ecol..

[45] Lung M, Espira A (2015). The influence of stand variables and human use on biomass and carbon stocks of a transitional African forest: Implications for forest carbon projects. For. Ecol. Manag..

[46] Bernard, C. Etude d’un parc à Prosopis africana au Nord Cameroun (cas du village de Holom, en pays Musey) : Premiers résultats. In *IRAD-Projet Garoua 11/CIRAD-Foret/ORSTOM/ICRAF*. 141 (1996).

[47] Grace JB (2016). Integrative modelling reveals mechanisms linking productivity and plant species richness. Nature.

[48] Nikiema, A. *Agroforestry Parkland Species Diversity : Uses and Management in Semi-Arid West Africa* (*Burkina Faso*) (2005).

[49] Vagen, T. G., Winowiecki, L. A., Tamene, L. & Tondoh, J. E. *Land Degradation Surveillance Framework* (*LSDF*): *Field Guide v4* (2013).

[50] Herrmann SM, Hutchinson CF (2005). The changing contexts of the desertification debate. J. Arid Environ..

[51] Karlson M, Ostwald M (2016). Remote sensing of vegetation in the Sudano-Sahelian zone: A literature review from 1975 to 2014. J. Arid Environ..

[52] Forkuor G (2020). Above-ground biomass mapping in West African dryland forest using Sentinel-1 and 2 datasets—A case study. Remote Sens. Environ..

[53] Faure P, Volkoff B (1998). Some factors affecting regional differentiation of the soils in the Republic of Benin (West Africa). Catena.

[54] Mbow C (2014). Agroforestry solutions to address food security and climate change challenges in Africa. Curr. Opin. Environ. Sustain..

[55] Bayen P (2020). Models for estimating aboveground biomass of four dryland woody species in Burkina Faso, West Africa. J. Arid Environ..

[56] Akoègninou, A., Burg, W. J. van der Maesen, L. J. G. *Flore Analytique du Bénin*. (Backhuys Publishers, 2006).

[57] Pérez-Harguindeguy N (2013). New handbook for standardised measurement of plant functional traits worldwide. Aust. J. Bot..

[58] Chave J (2014). Improved allometric models to estimate the aboveground biomass of tropical trees. Glob. Change Biol..

[59] Uscanga A, Bartlein PJ, Silva LCR (2023). Local and regional effects of land-use intensity on aboveground biomass and tree diversity in tropical Montane cloud forests. Ecosystems.

[60] Mensah S, Salako VK, Seifert T (2020). Structural complexity and large-sized trees explain shifting species richness and carbon relationship across vegetation types. Funct. Ecol..

[61] Dimobe K, Kuyah S, Dabré Z, Ouédraogo A, Thiombiano A (2019). Diversity-carbon stock relationship across vegetation types in W National Park in Burkina Faso. For. Ecol. Manag..

[62] Chabi A, Lautenbach S, Orekan VOA, Kyei-Baffour N (2016). Allometric models and aboveground biomass stocks of a West African Sudan Savannah watershed in Benin. Carbon Balance Manag..

[63] Nygård R, Elfving B (2000). Stem basic density and bark proportion of 45 woody species in young savanna coppice forests in Burkina Faso. Ann. For. Sci..

[64] Zanne, A. E. *et al.**Data from: Towards a Worldwide Wood Economics Spectrum*. *2047488 Bytes*. 10.5061/DRYAD.234 (2009). 10.1111/j.1461-0248.2009.01285.x 19243406

[65] Martin AR, Doraisami M, Thomas SC (2018). Global patterns in wood carbon concentration across the world’s trees and forests. Nat. Geosci..

[66] Laliberté E, Legendre P (2010). A distance-based framework for measuring functional diversity from multiple traits. Ecology.

[67] Villéger S, Mason NWH, Mouillot D (2008). New multidimensional functional diversity indices for a multifaceted framework in functional ecology. Ecology.

[68] Hsieh TC, Ma KH, Chao A (2016). iNEXT: An R package for rarefaction and extrapolation of species diversity (Hill numbers). Methods Ecol. Evol..

[69] Jost L (2006). Entropy and diversity. Oikos.

[70] Kattge J (2020). TRY plant trait database—Enhanced coverage and open access. Glob. Change Biol..

[71] Fern, K. *Useful Tropical Plants Database 2014*. https://www.feedipedia.org/node/21211 (2014).

[72] Maynard DS (2022). Global relationships in tree functional traits. Nat. Commun..

[73] Reich PB (2014). The world-wide ‘fast–slow’ plant economics spectrum: A traits manifesto. J. Ecol..

[74] Josse J, Husson F (2012). Handling missing values in exploratory multivariate data analysis methods. J. Soc. Franç. Stat..

[75] van der Plas F (2020). Plant traits alone are poor predictors of ecosystem properties and long-term ecosystem functioning. Nat. Ecol. Evol..

[76] Laliberté E, Legendre P, Shipley B (2015). Measuring functional diversity (FD) from multiple traits, and other tools for functional ecology. R Package Version.

[77] Ali A (2019). Climate and soils determine aboveground biomass indirectly via species diversity and stand structural complexity in tropical forests. For. Ecol. Manag..

[78] Hogg EH (1997). Temporal scaling of moisture and the forest-grassland boundary in western Canada. Agric. For. Meteorol..

[79] Poorter L (2017). Biodiversity and climate determine the functioning of Neotropical forests. Glob. Ecol. Biogeogr..

[80] Fick SE, Hijmans RJ (2017). WorldClim 2: New 1-km spatial resolution climate surfaces for global land areas. Int. J. Climatol..

[81] Leenaars, J. G. B., van Oostrum, A. J. M. & Ruiperez Gonzalez, M. *Africa Soil Profiles Database, Version 1.2. A Compilation of Georeferenced and Standardised Legacy Soil Profile Data for Sub-Saharan Africa (with Dataset).* 162 (2014).

[82] Bartoń K (2018). MuMIn: Multi-model inference. R Package Version.

[83] Jaeger B (2016). Package ‘r2glmm’. R Package.

[84] Bivand R, Wong DWS (2018). Comparing implementations of global and local indicators of spatial association. Test.

[85] Bivand RS, Pebesma E, Gomez-Rubio V (2013). Applied Spatial Data Analysis with R.

[86] Lefcheck JS (2016). piecewiseSEM: Piecewise structural equation modelling in r for ecology, evolution, and systematics. Methods Ecol. Evolut..

[87] Shipley B (2009). Confirmatory path analysis in a generalized multilevel context. Ecology.

[88] Nakagawa S, Schielzeth H (2013). A general and simple method for obtaining R2 from generalized linear mixed-effects models. Methods Ecol. Evolut..

[89] R Core Team. *R: A Language and Environment for Statistical Computing* (2020).

[90] GFW. *Global Forest Watch* (2014).

[91] Zomer RJ (2016). Global tree cover and biomass carbon on agricultural land: The contribution of agroforestry to global and national carbon budgets. Sci. Rep..

[92] Ruiz-Benito P (2014). Diversity increases carbon storage and tree productivity in Spanish forests. Glob. Ecol. Biogeogr..

[93] Wen Z, Jiang Z, Zheng H, Ouyang Z (2022). Tropical forest strata shifts in plant structural diversity-aboveground carbon relationships along altitudinal gradients. Sci. Total Environ..

[94] Tetemke BA, Birhane E, Rannestad MM, Eid T (2021). Species diversity and stand structural diversity of woody plants predominantly determine aboveground carbon stock of a dry Afromontane forest in Northern Ethiopia. Forest Ecol. Manag..

[95] Parker GG (2004). Three-dimensional structure of an old-growth Pseudotsuga-Tsuga canopy and its implications for radiation balance, microclimate, and gas exchange. Ecosystems.

[96] Ali A, Mattsson E (2017). Individual tree size inequality enhances aboveground biomass in homegarden agroforestry systems in the dry zone of Sri Lanka. Sci. Total Environ..

[97] Gnangle PC (2012). Perceptions locales du changement climatique et mesures d’adaptation dans la gestion des parcs à karité au Nord-Bénin. Int. J. Biol. Chem. Sci..

[98] Loreau M, Hector A (2001). Partitioning selection and complementarity in biodiversity experiments. Nature.

[99] Hitimana, J., Legilisho Kiyiapi, J. & Thairu Njunge, J. Forest structure characteristics in disturbed and undisturbed sites of Mt. Elgon Moist Lower Montane Forest, western Kenya. *For. Ecol. Manag.* **194**, 269–291 (2004).

[100] Bee JN, Kunstler G, Coomes DA (2007). Resistance and resilience of New Zealand tree species to browsing. J. Ecol..

[101] Hoffmann WA (1996). The effects of fire and cover on seedling establishment in a Neotropical Savanna. J. Ecol..

[102] Giglio L, Justice C, Boschetti L, Roy D (2017). NASA EOSDIS Land Process. DAAC.

[103] Kifle ET, Noulèkoun F, Son Y, Khamzina A (2022). Woody species diversity, structural composition, and human use of church forests in central Ethiopia. For. Ecol. Manag..

