# Supplementary material for: Tree size diversity is the major driver of aboveground carbon storage in dryland agroforestry parklands
Source: Sci Rep. 2023 Dec 14;13:22210. doi: 10.1038/s41598-023-49119-9 (PMC10721610; doi:10.1038/s41598-023-49119-9)
Supplement: Supplementary file 1 — Supplementary Information. [file 41598_2023_49119_MOESM1_ESM.docx]

**Supplementary Information**

**Tree size diversity is the major driver of aboveground carbon stock in dryland agroforestry parklands**

Florent Noulèkoun^1^, Sylvanus Mensah^2,3^, HyungSub Kim^4^, Heejae Jo^1,4^, Gerard N. Gouwakinnou^5^, Thierry D. Houehanou^5^, Michael Mensah^6^, Jesse Naab^7^, Yowhan Son^4^, Asia Khamzina^1^*

**Supplementary Methods: calculation of crown area, species diversity attributes and Importance Value Index (IVI)**

The crown area of individual trees was calculated as follow (Bayen et al., 2020):

| $CA= \pi x \left( \frac{d_{1}}{2} \right) x \left( \frac{d_{2}}{2} \right)$ | (Eq. 1) |
| --- | --- |

where CA is the crown area (m^2^) and d_1_ and d_2_ are the vertical north-south and east-west projections of the crown on the ground with d_1_ perpendicular to d_2_.

We used the Shannon species diversity (H_SR_, Eq. 2) to characterize taxonomic diversity. The H_SR_ was computed using the “ChaoShannon” from the R package “iNEXT” (Hsieh et al., 2016):

| $H_{SR}= -\sum_{i=1}^{s} p_{i} \times{\log p}_{i}$ | (Eq. 2) |
| --- | --- |

where H_SR_ is the Shannon species diversity, s is the species richness and p_i_ is the relative basal area of i^th^ species.

The functional identity was quantified using the community-weighted mean (CWM) of functional traits (Eq. 3). The CWM of traits is the mean values of traits of species present in the community weighted by their relative abundance (Lavorel et al., 2008) and was calculated for each plot as follows:

| $CMW=\sum_{i=1}^{s} p_{i} T_{i}$ | (Eq. 3) |
| --- | --- |

where “s” is the species richness within a plot and pi and Ti represent the relative basal area and the trait value of the ith species in each plot, respectively. Both F_dis_ and CWM of traits were computed using the R-package “FD” (Laliberté et al., 2015).

The structural diversity was characterized using the Shannon structural diversity index (H, Eq. 4 and 5) and the coefficient of variation (CV, Eq. 6 and 7) of tree DBH and height. H was calculated for DBH and height as follows (Staudhammer and LeMay, 2011):

| $H_{DBH}= -\sum_{i=1}^{d} p_{i} ln(p_{i})$ | (Eq. 4) |
| --- | --- |
| $H_{height}= -\sum_{j=1}^{h} p_{j} ln(p_{j})$ | (Eq. 5) |

where pi and pj are the basal area as a proportion of the total plot basal area of all individuals in the ith DBH class and jth height class, respectively, and d and h represent the total number of DBH and height classes, respectively. H_DBH_ was calculated for discrete DBH classes of 5, 10 and 15, whereas the H_heigh_ values were calculated considering classes of 4, 8 and 12.

The coefficients of variation (CV) of DBH and height were calculated as follows:

| ${CV}_{DBH}=\frac{{SD}_{DBH}}{\bar{X}_{DBH}}$ | (Eq. 6) |
| --- | --- |
| ${CV}_{height}=\frac{{SD}_{height}}{\bar{X}_{height}}$ | (Eq. 7) |

where ${SD}_{DBH}$ and ${SD}_{height}$ are the standard deviations of DBH and height for all individual stems within the plot, respectively, and $\bar{X}_{DBH}$ and $\bar{X}_{height}$represent the plot average DBH and height of all individual stems, respectively.

The Importance Value Index (IVI; Curtis and McIntosh, 1951) was computed in the R package “BidoiversityR” based on the following formula (Tadesse Kifle et al., 2022):

| $\mathrm{IVI}=RFr+RDe+RDo$ | (Eq. 8) |
| --- | --- |

| $RFr=\frac{f_{i}}{\sum_{i=1}^{s} f_{i}}$ | (Eq. 9) |
| --- | --- |

| $\mathrm{RDe}=\frac{n_{i}}{\sum_{i=1}^{s} n_{i}}$ | (Eq. 10) |
| --- | --- |

| $RDo=\frac{c_{i}}{\sum_{i=1}^{s} c_{i}}$ | (Eq. 11) |
| --- | --- |

where RFr, RDe and RDo are the relative frequency, relative density and relative dominance, respectively; ni, fi, and ci are the density (i.e., total number of individuals of a species), frequency (i.e., the number of plots where a species is observed divided by the total number of plots), and basal area (i.e., total basal area of a species) of the ith species, respectively, and *s* is the total number of species recorded.

**Tables**

**Table S1.** Descriptive statistics of all the variables used in this study. All the values presented here are non-transformed (i.e., original data). Standardized values were used in the statistical analyses.

| Variable (per group) | Unit | Mean | S.D. | Minimum | Maximum |
| --- | --- | --- | --- | --- | --- |
| Human management | | | | | |
| Density of standing woody species | Trees ha^-1^ | 72.75 | 42.95 | 20.00 | 220.00 |
| Number of harvested trees (NHT)^1^ | Trees plot^-1^ | 13.43 | 22.14 | 0.00 | 132.00 |
| Abiotic | | | | | |
| Climate | | | | | |
| Mean Annual Precipitation (MAP) | mm | 1022.00 | 44.54 | 917.00 | 1127.00 |
| Mean annual potential evapotranspiration (ETPo) | mm | 2242.00 | 121.12 | 1810.00 | 2388.00 |
| Climatic moisture index (CMI)^2^ | mm year^-1^ | -1220.00 | 150.27 | -1449.00 | -740.00 |
| Aridity Index (AI) | - | 0.46 | 0.04 | 0.39 | 0.60 |
| Topography | | | | | |
| Elevation | m | 240.21 | 80.71 | 146.00 | 483.00 |
| Soil | | | | | |
| Clay content | % | 26.21 | 1.86 | 21.75 | 30.28 |
| Silt content | % | 22.54 | 2.71 | 17.33 | 27.55 |
| Sand content | % | 50.93 | 3.88 | 43.12 | 59.97 |
| Cation exchange capacity (CEC) | cmol kg^-1^ | 9.53 | 2.07 | 6.08 | 18.00 |
| Total organic carbon content (C%) | % | 0.55 | 0.29 | 0.22 | 2.01 |
| Total nitrogen content (N%) | % | 0.14 | 0.11 | 0.03 | 0.50 |
| Biotic | | | | | |
| Taxonomic diversity | | | | | |
| Shannon diversity (H_SR_)^3^ | - | 1.49 | 0.52 | 0.62 | 3.17 |
| Functional diversity |  |  |  |  |  |
| Functional richness (F_rich_) | - | 3.51 | 4.09 | 0.00 | 16.11 |
| Functional evenness (F_eve_) | - | 0.48 | 0.31 | 0.00 | 0.99 |
| Functional divergence (F_div_) | - | 0.62 | 0.35 | 0.00 | 0.98 |
| Functional dispersion (F_dis_) | - | 2.51 | 1.05 | 0.35 | 4.72 |
| Functional identity | | | | | |
| Community-weighted mean of wood density (CWM_ρ_) | g∙cm^-3^ | 0.63 | 0.14 | 0.33 | 0.90 |
| Community-weighted mean of maximum tree height (CWM_MAXH_) | m | 17.60 | 3.70 | 8.18 | 22.91 |
| Community-weighted mean of specific leaf area (CWM_SLA_) | mm^2^ mg^-1^ | 11.86 | 2.57 | 6.71 | 16.59 |
| Community-weighted mean of leaf area (CWM_LA_) | cm^2^ | 43.33 | 22.44 | 3.83 | 114.53 |
| Community-weighted mean of leaf carbon content (CWM_LC%_) | % | 42.78 | 3.08 | 34.55 | 46.86 |
| Community-weighted mean of leaf nitrogen content (CWM_LN%_) | % | 1.29 | 0.20 | 0.91 | 1.82 |
| Community-weighted mean of leaf dry matter content (CWM_LDMC_) | mg g^-1^ | 356.70 | 51.93 | 254.83 | 466.02 |
| Community-weighted mean of leaf habit (CWM_LH_)^4^ | - | - | - | 0.00 | 1.00 |
| Community-weighted mean of nitrogen fixation (CWM_NF_)^5^ | - | - |  | 0.00 | 1.00 |
| Community-weighted mean of maximum crown area (CWM_MAXCA_) | m^2^ | 262.40 | 163.73 | 31.20 | 616.71 |
| Structural diversity | | | | | |
| Shannon structural diversity index for 5 cm DBH bin (H_DBH5_) | - | 1.27 | 0.46 | 0.15 | 2.19 |
| Shannon structural diversity index for 10 cm DBH bin (H_DBH10_) | - | 1.10 | 0.46 | 0.15 | 2.01 |
| Shannon structural diversity index for 15 cm DBH bin (H_DBH15_) | - | 1.01 | 0.42 | 0.00 | 1.86 |
| Shannon structural diversity index for 4 m height bin (H_h4_) | - | 0.75 | 0.36 | 0.00 | 1.36 |
| Shannon structural diversity index for 8 m height bin (H_h8_) | - | 0.52 | 0.31 | 0.00 | 1.25 |
| Shannon structural diversity index for 12 m height bin (H_h12_) | - | 0.34 | 0.31 | 0.00 | 1.09 |
| Coefficient of variation of DBH (CV_DBH_) | - | 51.72 | 26.48 | 12.33 | 134.77 |
| Coefficient of variation of height (CV_h_) | - | 34.63 | 15.01 | 9.81 | 65.13 |
| Coefficient of variation of crown area (CV_CA_) | - | 83.69 | 41.34 | 13.69 | 195.86 |
| Ecosystem function | | | | | |
| Aboveground carbon (AGC) stock | Mg ha^-1^ | 82.26 | 124.74 | 0.64 | 585.55 |

^1^The NHT included all individual trees and shrubs that were either pruned or cut down per plot.

^2^CMI was multiplied by – 1 for the statistical analyses.

^3^The Shannon species diversity (H_SR_) was estimated using “ChaoShannon” function of the R package “iNEXT” to account for species rarefaction and variation in sampling efforts (Hsieh et al., 2016; Jost, 2006).

^4^The leaf habit relates to the deciduous and evergreen status of a tree. A deciduous species was assigned a value of 1 and an evergreen species was assigned a value of 0 (Hisano and Chen, 2020).

^5^The nitrogen fixation status relates to the ability of a species to fix atmospheric nitrogen or not. A non-fixing species was assigned a value of 0 whereas a nitrogen-fixing species was assigned a value of 1.

**Table S2.** Characteristics of the woody species enumerated in the parklands. The species were ranked according to their Importance Value Index (IVI). SD: standard deviation.

| Species name | Family | Life form^1^ | Leaf habit^1^ | Nitrogen fixation^1^ | Density^2^ | IVI^3^ | DBH  (Mean + SD) | Height  (Mean + SD) |
| --- | --- | --- | --- | --- | --- | --- | --- | --- |
| *Vitellaria paradoxa* C.F.Gaertn. | Sapotaceae | Tree | Deciduous | Non-fixer | 90 | 61.69 | 62.97 + 44.32 | 8.77 + 2.74 |
| *Parkia biglobosa* (Jacq.) R.Br. ex G.Don | Fabaceae | Tree | Deciduous | Fixer | 42 | 43.24 | 78.20 + 70.13 | 11.11 + 4.93 |
| *Adansonia digitata* L. | Malvaceae | Tree | Deciduous | Non-fixer | 21 | 23.96 | 110.95 + 61.55 | 13.36 + 4.79 |
| *Azadirachta indica* A.Juss. | Meliaceae | Tree | Evergreen | Non-fixer | 26 | 12.74 | 18.93 + 12.93 | 7.39 + 2.44 |
| *Lannea microcarpa* Engl. & K.Krause | Anacardiaceae | Tree | Deciduous | Non-fixer | 17 | 11.96 | 43.49 + 20.38 | 7.68 + 2.01 |
| *Daniella oliveri* (Rolfe) Hutch. & Dalziel | Fabaceae | Tree | Deciduous | Non-fixer | 5 | 11.56 | 199.80 + 65.28 | 16.84 + 1.76 |
| *Borassus aethiopum* Mart. | Arecaceae | Palm | Evergreen | Non-fixer | 17 | 10.56 | 56.61 + 29.16 | 10.41 + 3.27 |
| *Bombax costatum* Pellegr. & Vuillet. | Malvaceae | Tree | Deciduous | Non-fixer | 9 | 7.98 | 64.44 + 63.45 | 8.77 + 5.82 |
| *Terminalia microcarpa* Decne | Combretaceae | Tree | Semi-deciduous | Non-fixer | 9 | 7.91 | 84.65 + 44.64 | 10.63 + 5.26 |
| *Eucalyptus camaldulensis* Dehnh. | Myrtaceae | Tree | Evergreen | Non-fixer | 10 | 6.26 | 54.04 + 58.85 | 12.00 + 4.69 |
| *Combretum fragrans* F. Hoffm. | Combretaceae | Tree | Deciduous | Non-fixer | 11 | 6.15 | 54.45 + 25.06 | 6.87 + 2.24 |
| *Afzelia africana* Sm. Ex Pers. | Fabaceae | Tree | Deciduous | Non-fixer | 6 | 5.45 | 56.68 + 43.26 | 9.32 + 3.88 |
| *Terminalia mollis* M.A.Lawson | Combretaceae |  | Deciduous | Non-fixer | 8 | 4.99 | 24.50 + 8.81 | 7.65 + 2.23 |
| *Diospyros mespiliformis* Hochst. Ex A. DC. | Ebenaceae | Tree | Evergreen | Non-fixer | 5 | 4.90 | 77.63 + 85.54 | 8.55 + 2.65 |
| *Sterculia setigera* Delile | Malvaceae | Tree | Deciduous | Non-fixer | 3 | 4.78 | 137.40 + 78.52 | 12.37 + 4.08 |
| Ficus sycomorus L. | Moraceae | Tree | Semi-deciduous | Non-fixer | 6 | 4.45 | 51.10 + 19.62 | 8.83 + 2.89 |
| *Senegalia gourmaensis* (A.Chev.) Kyal. & Boatwr. | Fabaceae | Shrub/  Tree | Deciduous | Fixer | 8 | 4.24 | 38.21 + 11.66 | 7.27 + 2.51 |
| *Mitragyna inermis* (Willd.) Kuntze | Rubiaceae | Shrub/  Tree | Evergreen | Non-fixer | 5 | 3.87 | 42.73 + 16.16 | 4.73 + 0.78 |
| *Combretum lecardii* Engl. & Diels | Combretaceae | Shrub | Deciduous | Non-fixer | 6 | 3.79 | 39.56 + 32.37 | 5.59 + 1.94 |
| *Ficus ovata* Vahl | Moraceae | Shrub/  Tree | Evergreen | Non-fixer | 4 | 3.79 | 53.33 + 32.47 | 9.91 + 5.53 |
| *Tamarindus indica* L. | Fabaceae | Tree | Evergreen | Fixer | 4 | 3.76 | 91.28 + 42.36 | 9.43 + 3.25 |
| *Sarcocephalus latifolius* Sm. | Rubiaceae | Shrub | Deciduous | Non-fixer | 5 | 3.73 | 36.58 + 8.21 | 4.91 + 1.94 |
| *Anacardium occidentale* L. | Anacardiaceae | Shrub/  Tree | Evergreen | Non-fixer | 4 | 3.39 | 30.55 + 21.96 | 4.96 + 1.03 |
| *Isoberlinia doka* Craib & Stapf | Fabaceae | Tree | Evergreen | Non-fixer | 5 | 3.34 | 52.68 + 46.50 | 8.43 + 2.55 |
| *Monotes kerstingii* Gilg | Dipterocarpaceae | Shrub/  Tree | - | Non-fixer | 5 | 3.11 | 56.65 + 16.70 | 6.37 + 2,04 |
| *Ficus exasperata* Vahl | Moraceae | Tree | Deciduous | Non-fixer | 3 | 2.72 | 47.60 + 24.49 | 8.58 + 3.62 |
| *Vitex doniana* Sweet | Lamiaceae | Tree | Deciduous | Fixer | 3 | 2.58 | 36.02 + 20.86 | 7.82 + 3.18 |
| *Burkea africana* Hook. | Fabaceae | Shrub/  Tree | Deciduous | Non-fixer | 4 | 2.53 | 69.00 + 33.11 | 10.66 + 3.64 |
| *Ficus dicranostyla* Mildbr. | Moraceae | Shrub/  Tree | Evergreen | Non-fixer | 4 | 2.48 | 41.70 + 22.68 | 8.31 + 4.54 |
| *Lannea acida* A.Rich. | Anacardiaceae | Shrub/  Tree | Deciduous | Non-fixer | 2 | 2.30 | 90.00 + 25.46 | 12.57 + 2.60 |
| *Khaya senegalensis* (Desr.) A.Juss. | Meliaceae | Tree | Evergreen | Non-fixer | 3 | 2.27 | 40.62 + 49.29 | 7.26 + 3.97 |
| *Prosopis africana* (Guill. & Perr.) Taub. | Fabaceae | Tree | Deciduous | Fixer | 2 | 2.13 | 64.20 + 67.60 | 6.68 + 2.72 |
| *Piliostigma thonningii* (Schumach.) Milne-Redh. | Fabaceae | Shrub/  Tree | Semi-deciduous | Fixer | 3 | 1.94 | 22.63 + 16.17 | 4.07 + 0.17 |
| *Combretum collinum* Fresen. | Combretaceae | Shrub/  Small tree | Deciduous | Non-fixer | 2 | 1.92 | 62.00 + 12.73 | 9.64 + 0.23 |
| *Grewia venusta* Fresen. | Malvaceae | Shrub/  Small tree | Deciduous | Non-fixer | 2 | 1.80 | 49.00 + 16.97 | 6.23 + 1.00 |
| *Zanthoxylum zanthoxyloides* (Lam.) Zepern. & Timler | Rutaceae | Shrub | Deciduous | Non-fixer | 2 | 1.61 | 13.66 + 7.86 | 4.12 + 0.52 |
| *Hexalobus monopetalus* (A.Rich.) Engl. & Diels | Annonaceae | Shrub/  Small tree | Semi-deciduous | Non-fixer | 2 | 1.06 | 6.60 + 0.14 | 3.39 + 0.36 |
| *Terminalia albida* Scott-Elliot | Combretaceae | Tree | Deciduous | Non-fixer | 1 | 1.05 | 78.00 | 9.13 |
| *Isoberlinia tomentosa* (Harms) Craib & Stapf | Fabaceae | Tree | Evergreen | Fixer | 1 | 1.03 | 75.00 | 9.01 |
| *Crossopteryx febrifuga* (Afzel. ex G.Don) Benth. | Rubiaceae | Tree | Deciduous | Non-fixer | 1 | 0.97 | 64.00 | 6.82 |
| *Sclerocarya birrea* (A.Rich.) Hochst. | Anacardiaceae | Tree | Deciduous | Non-fixer | 1 | 0.91 | 52.10 | 12.43 |
| *Sapium ellipticum* (Hochst) Pax | Euphorbiaceae | Tree | Semi-deciduous | Non-fixer | 1 | 0.90 | 51.00 | 4.38 |
| *Blighia sapida* K.D.Koenig | Sapindaceae | Tree | Evergreen | Non-fixer | 1 | 0.88 | 44.10 | 11.56 |
| *Phoenix dactylifera* L. | Arecaceae | Palm | Evergreen | Non-fixer | 1 | 0.84 | 32.50 | 5.53 |
| *Lannea velutina* A.Rich. | Anacardiaceae | Shrub/  Tree | Deciduous | Non-fixer | 1 | 0.82 | 26.60 | 8.37 |
| *Ficus glumosa* Delile | Moraceae | Tree | Semi-deciduous | Non-fixer | 1 | 0.82 | 26.40 | 7.74 |
| *Terminalia avicennioides* Guill. & Perr. | Combretaceae | Shrub/ Small tree | - | Non-fixer | 1 | 0.82 | 25.20 | 6.77 |
| *Vachellia sieberiana* (DC.) Kyal. & Boatwr. | Fabaceae | Tree | Deciduous | Fixer | 1 | 0.82 | 22.60 | 4.45 |
| *Strychnos spinosa* Lam. | Loganiaceae | Tree | Deciduous | Non-fixer | 1 | 0.81 | 22.00 | 3.72 |
| *Vachellia seyal* (Delile) P.Hurter | Fabaceae | Tree | Evergreen | Fixer | 1 | 0.80 | 12.87 | 3.56 |
| *Detarium microcarpum* Guill. & Perr. | Fabaceae | Shrub/  Small tree | Deciduous | Fixer | 1 | 0.80 | 9.50 | 5.28 |
| *Vachellia nilotica* (L.) P.Hurter & Mabb. | Fabaceae | Tree | Evergreen | Fixer | 1 | 0.79 | 4.55 | 2.80 |

^1^The life form and leaf habit of the species were extracted from the “Useful Tropical Plants” database (Fern, 2014).

^2^Density represents the total number of individuals of a species across the 51 sampling plots.

^3^The calculation of the IVI is described in the “Methods” section above (Eq. 8, 9, 10 and 11).

**Table S3.** Relative importance of soil, functional diversity, functional identity, and structural diversity used as predictors of AGC stocks in the multi-model inferences analyses. Bold values indicate the most representative variable per group of variables, which was used in subsequent analyses. Acronyms are as described in Table S1.

| Variable grouping | Predictor variable | Relative importance |
| --- | --- | --- |
| Soil | Clay content | 0.23 |
|  | Silt content | 0.21 |
|  | Sand content | - |
|  | CEC | 0.27 |
|  | **C%** | **0.91** |
|  | N% | 0.90 |
| Functional diversity | F_rich_ | 0.44 |
|  | F_eve_ | 0.32 |
|  | **F_div_** | **0.46** |
|  | F_dis_ | 0.37 |
| Functional identity | CWM_ρ_ | 0.22 |
|  | **CWM_MAXH_** | **0.98** |
|  | CWM_SLA_ | - |
|  | CWM_LA_ | 0.62 |
|  | CWM_LC%_ | 0.22 |
|  | CWM_LN%_ | 0.26 |
|  | CWM_LDMC_ | 0.29 |
|  | CWM_LH_ | 0.31 |
|  | CWM_NF_ | 0.37 |
|  | CWM_MAXCA_ | - |
| Structural diversity | H_DBH5_ | 0.24 |
|  | H_DBH10_ | - |
|  | **H_DBH15_** | **1.00** |
|  | H_h4_ | - |
|  | H_h8_ | 0.26 |
|  | H_h12_ | 0.75 |
|  | CV_DBH_ | 0.22 |
|  | CV_h_ | 0.24 |
|  | CV_CA_ | 0.22 |

“-” indicates variables that were not retained in the final model owing to their high VIF values (>5).

**Table S4.** Results of the multiple linear mixed-effects model (LMM), assessing the direct effects of multiple predictor variables on aboveground carbon (AGC) stocks in agroforestry parklands (Fig. 3). The standardized coefficient, variance inflation factor (VIF) and relative contribution of the predictor variables are shown. The proportion of variance explained by both the fixed and random factors (total pseudo R^2^) was 78%. The relative contribution (partial pseudo R^2^) of the predictor variables was normalized to sum to 100%. The Shapiro–Wilk test showed that the model’s residuals were normally distributed (w = 0.989, p = 0.943). The Moran’s I test also indicated that the model’s residuals were not spatially auto-correlated (observed Moran’s I = -0.05, p = 0.084). SE: standard error. Other acronyms are described in Table 1. Significant effects are emboldened.

| Variable category | | Predictor variable | Estimate | SE | p-value | VIF | RC (%) | |
| --- | --- | --- | --- | --- | --- | --- | --- | --- |
| Human management | Human management | NHT | 0.03 | 0.08 | 0.703 | 1.10 | 0.37 | 0.19 |
| Abiotic factors | Climate | CMI | 0.09 | 0.13 | 0.517 | 2.07 | 1.30 | 18.99 |
|  | Topography | Elevation | 0.20 | 0.11 | 0.088 | 2.16 | 6.33 |  |
|  | **Soil** | **C%** | **0.21** | **0.09** | **0.023** | **1.41** | **11.36** |  |
| Biotic factors | Taxonomic diversity | H_SR_ | -0.12 | 0.11 | 0.258 | 2.01 | 2.79 | 80.82 |
|  | Functional diversity | F_div_ | 0.02 | 0.10 | 0.832 | 1.94 | 0.19 |  |
|  | **Functional identity** | **CWM_MAXH_** | **0.32** | **0.09** | **0.001** | **1.37** | **23.84** |  |
|  | **Structural diversity** | **H_DBH_** | **0.59** | **0.10** | **0.000** | **1.78** | **53.82** |  |

**Table S5.** Standardized effects of predictor and mediator variables on aboveground carbon (AGC) stocks based on the output of the piecewise structural equation model (pSEM) displayed in Fig. 4. Significant effects (p < 0.05) are indicated in bold. Acronyms are described in Table 1.

| Pathway | Standardized coefficients | S.E. | P-value |
| --- | --- | --- | --- |
| ***Direct effects of predictor and mediator variables*** | | | |
| H_SR_ ← NHT | -0.06 | 0.138 | 0.492 |
| **H_SR_ ← CMI** | **-0.379** | **0.183** | **0.044** |
| H_SR_ ← C% | 0.089 | 0.145 | 0.545 |
| H_SR_ ← Elevation | -0.008 | 0.173 | 0.965 |
| F_div_ ← NHT | 0.210 | 0.129 | 0.110 |
| F_div_ ← CMI | 0.091 | 0.177 | 0.610 |
| F_div_ ← C% | 0.183 | 0.135 | 0.182 |
| F_div_ ← Elevation | -0.151 | 0.160 | 0.352 |
| **F_div_ ← H_SR_** | **0.509** | **0.136** | **<0.001** |
| CWM_MAXH_ ← NHT | 0.010 | 0.141 | 0.943 |
| CWM_MAXH_ ← CMI | -0.173 | 0.195 | 0.378 |
| CWM_MAXH_ ← C% | -0.153 | 0.148 | 0.307 |
| CWM_MAXH_ ← Elevation | 0.188 | 0.176 | 0.292 |
| **CWM_MAXH_ ← H_SR_** | **-0.346** | **0.150** | **0.025** |
| H_DBH_ ← NHT | 0.166 | 0.127 | 0.200 |
| H_DBH_ ← CMI | 0.123 | 0.176 | 0.487 |
| **H_DBH_ ← C%** | **0.298** | **0.134** | **0.031** |
| **H_DBH_ ← Elevation** | **0.454** | **0.159** | **0.007** |
| H_DBH_ ← H_SR_ | 0.043 | 0.135 | 0.755 |
| AGC ← NHT | 0.031 | 0.081 | 0.703 |
| AGC ← CMI | 0.085 | 0.129 | 0.512 |
| **AGC ← C%** | **0.210** | **0.089** | **0.023** |
| AGC ← Elevation | 0.196 | 0.112 | 0.089 |
| AGC ← H_SR_ | -0.123 | 0.107 | 0.258 |
| AGC ← F_div_ | 0.022 | 0.104 | 0.832 |
| **AGC ← CWM_MAXH_** | **0.315** | **0.088** | **<0.001** |
| **AGC ← H_DBH_** | **0.591** | **0.099** | **<0.001** |
| C% ← NHT | -0.067 | 0.143 | 0.643 |
| ***Covariance effects*** | | | |
| **H_DBH_ ↔ F_div_** | **0.411** | **-** | **0.002** |
| **CWM_MAXH_ ↔ F_div_** | **0.363** | **-** | **0.005** |
| **C% ↔ CMI** | **-0.364** | **-** | **0.005** |
| ***R^2^ values for the endogenous variables*** | |  | ***Conditional R^2^*** |
| H_SR_ | |  | 0.16 |
| F_div_ | |  | 0.28 |
| CWM_MAXH_ | |  | 0.15 |
| H_DBH_ | |  | 0.29 |
| AGC | |  | 0.78 |
| C% | |  | 0.00 |

**Figures**

| 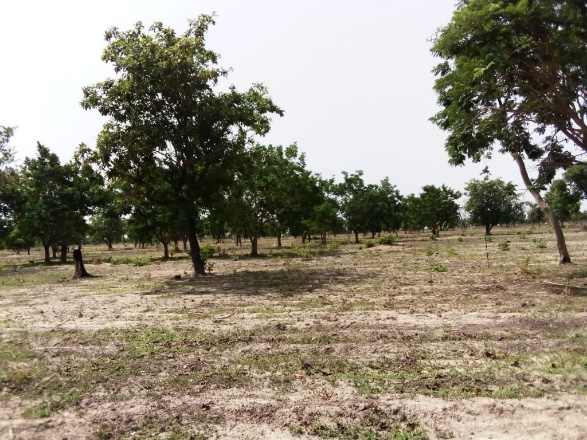 | 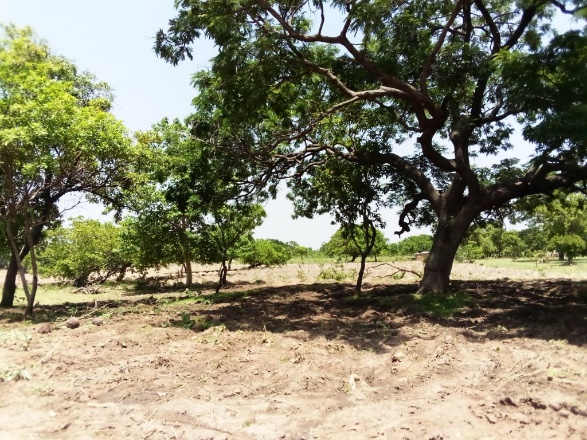 |
| --- | --- |

**Fig. S1.** Images of agroforestry parklands in the northern Benin.

| 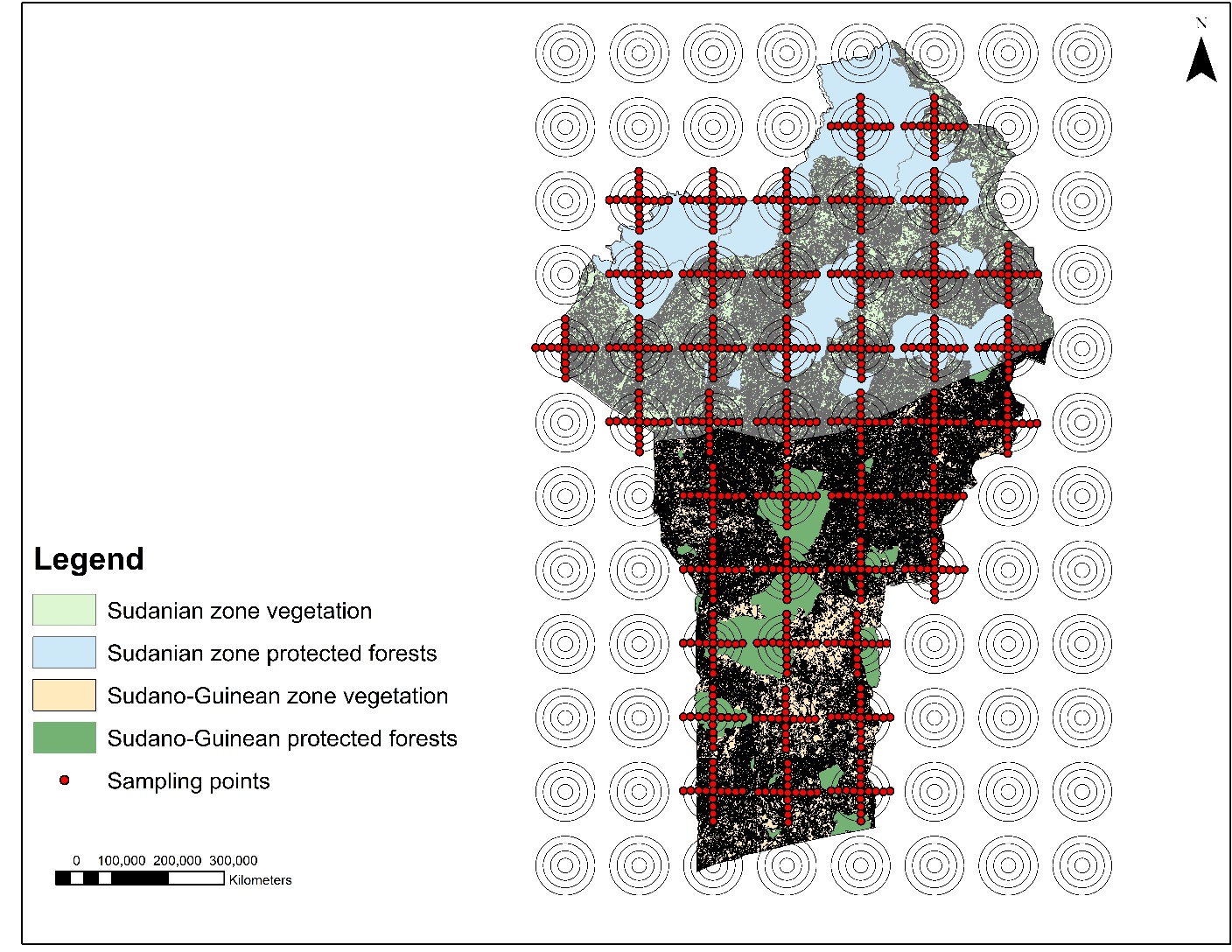 |
| --- |

**Fig. S2.** Layout of the sampling plots in the Sudanian and Sudano-Guinean zones of Benin. The distribution of the sampling points on the four concentric circles of 5, 10, 15 and 20 km are displayed. Each set of concentric circles is located within a grid of 50 km x 50 km (2500 km^2^).

| 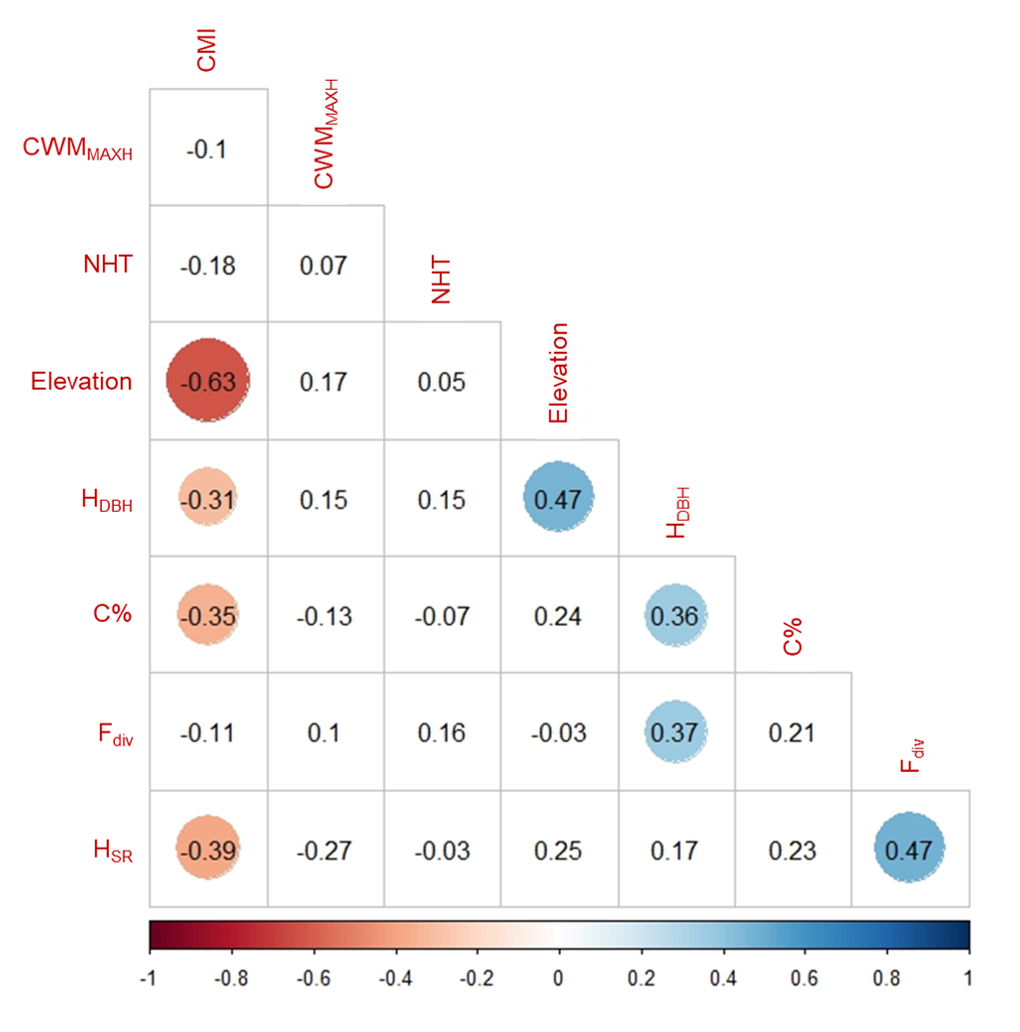 |
| --- |

**Fig. S3.** Pearson’s correlation coefficient between all pairs of selected abiotic and biotic predictors of aboveground carbon (AGC) stocks, which were used in the multiple mixed-effects regression models and piecewise structural equation model (pSEM; Fig. 3, 4 and 5). The squares with shaded circles inside represent significant correlations and the size of the circles is indicative of the magnitude of the correlation coefficient. Values represent the correlation coefficients. Acronyms are described in Table S1.

| 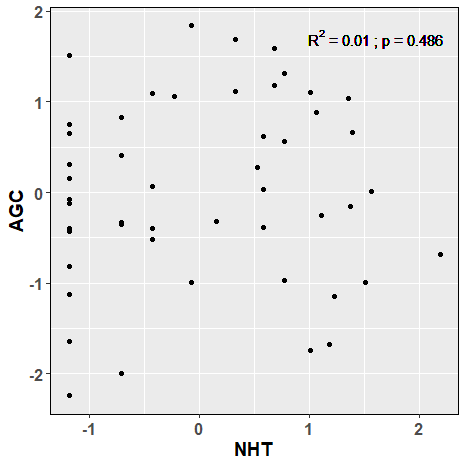 | 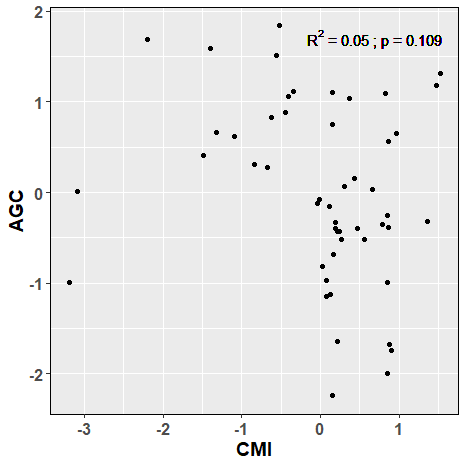 | 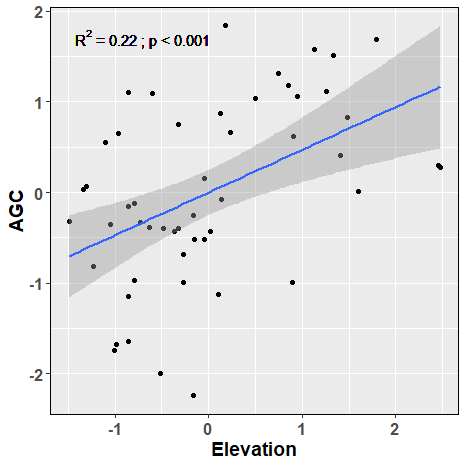 | 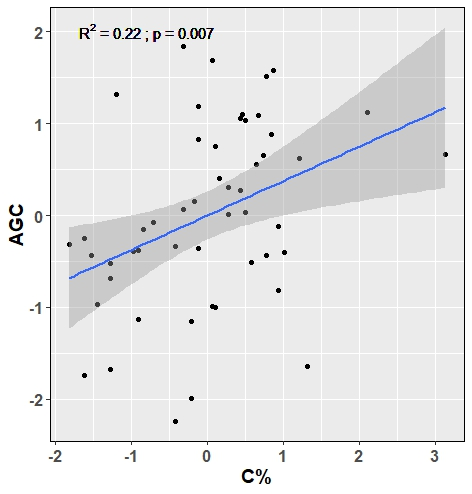 |
| --- | --- | --- | --- |
| 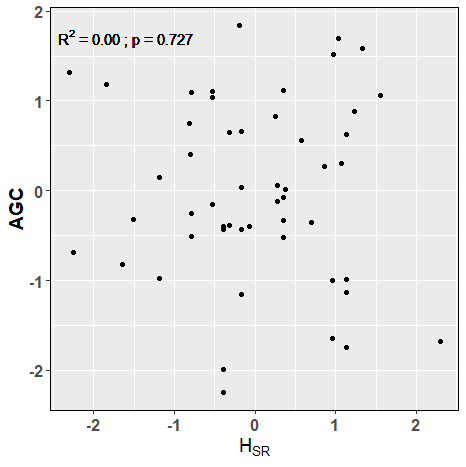 | 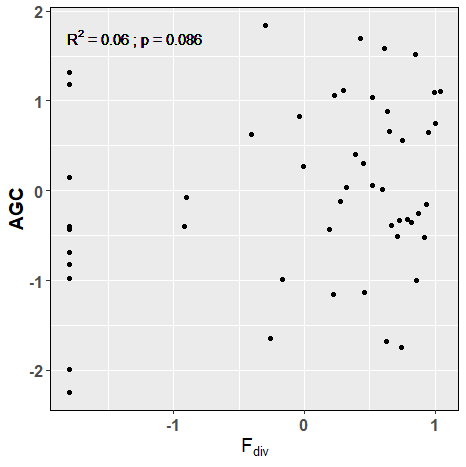 | 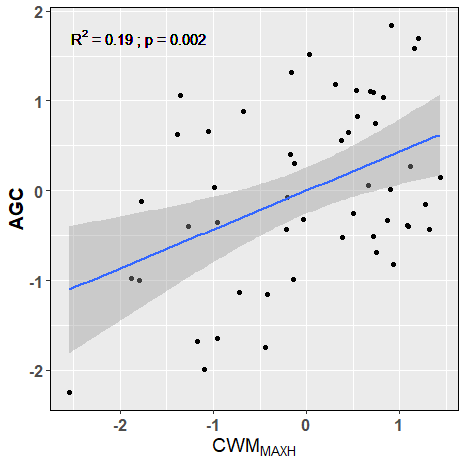 | 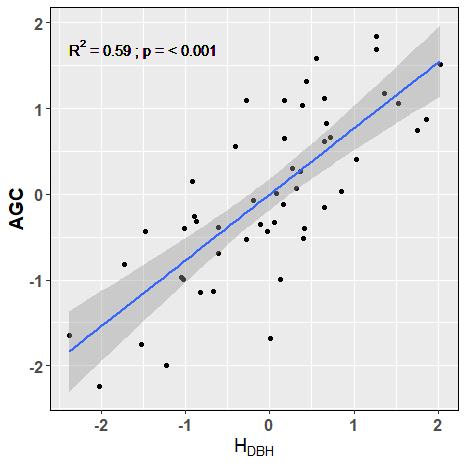 |
| 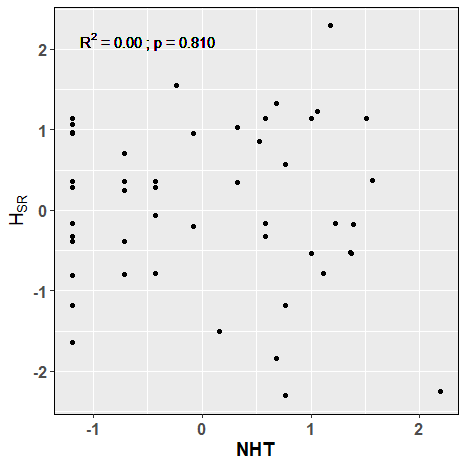 | 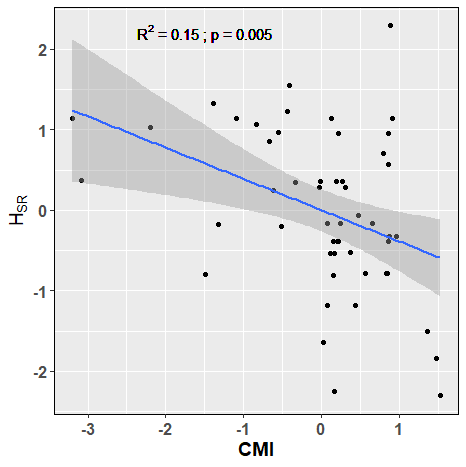 | 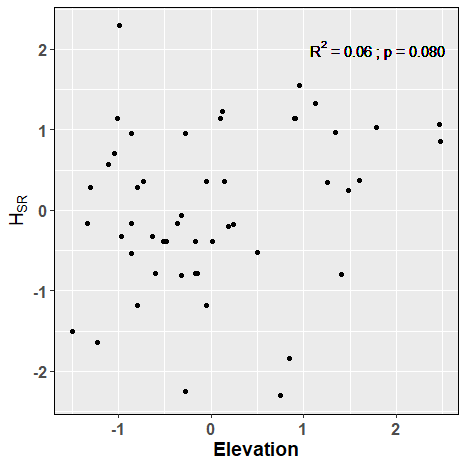 | 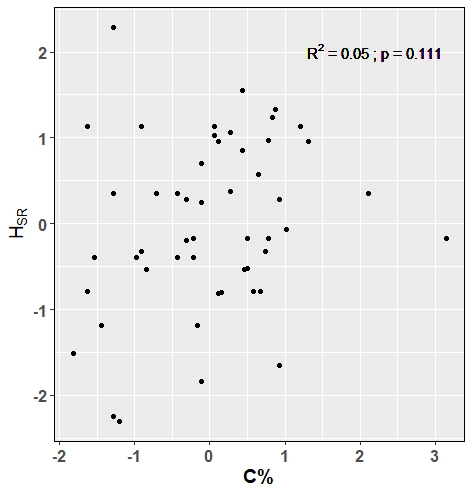 |
| 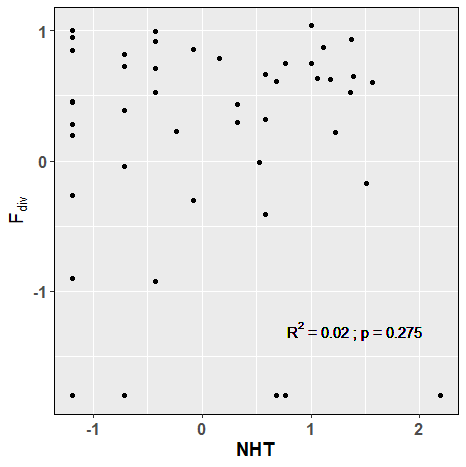 | 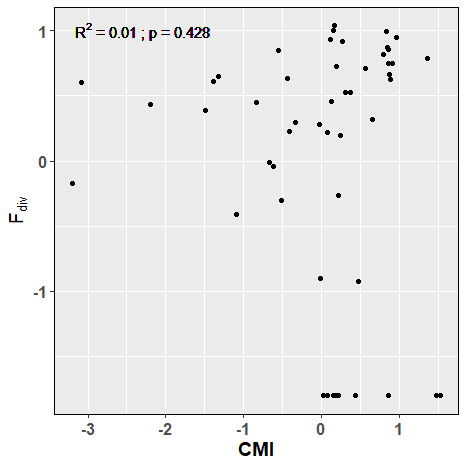 | 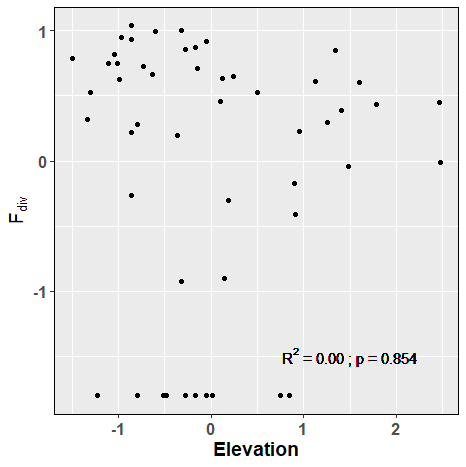 | 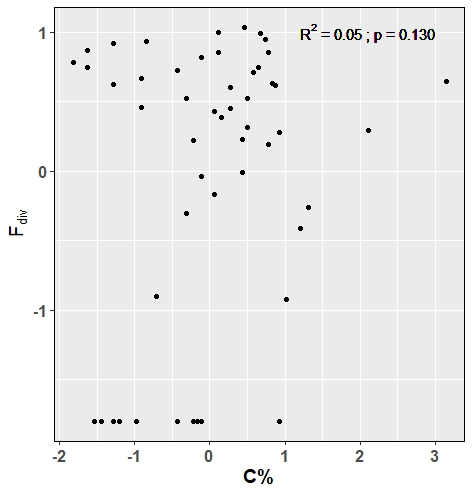 |
| 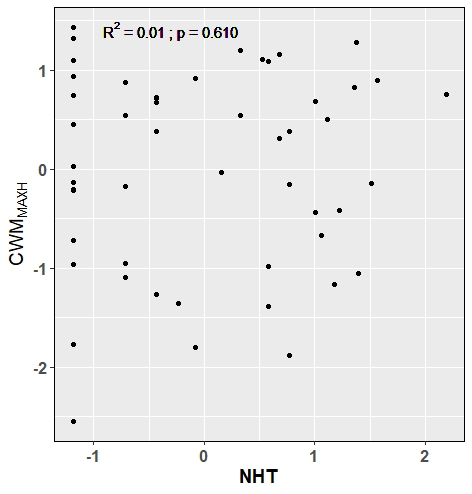 | 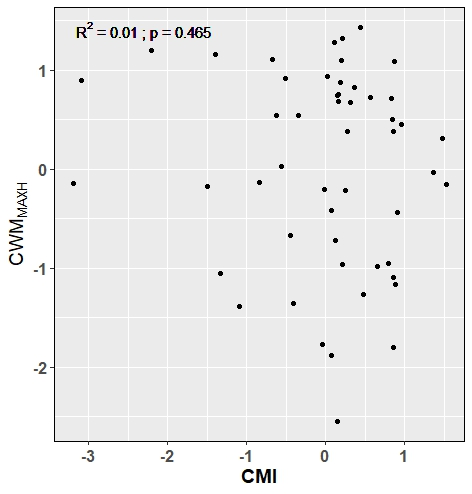 | 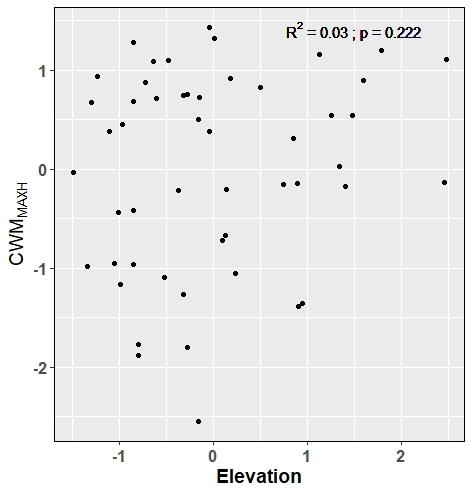 | 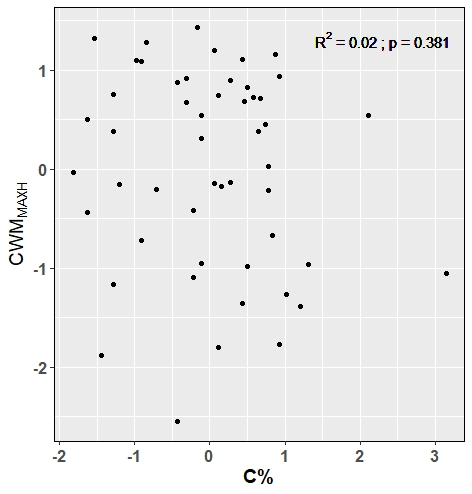 |
| 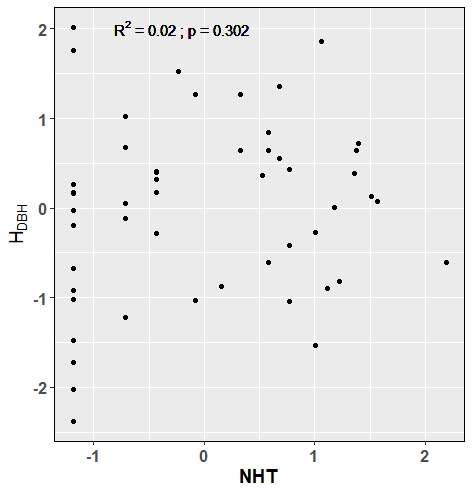 | 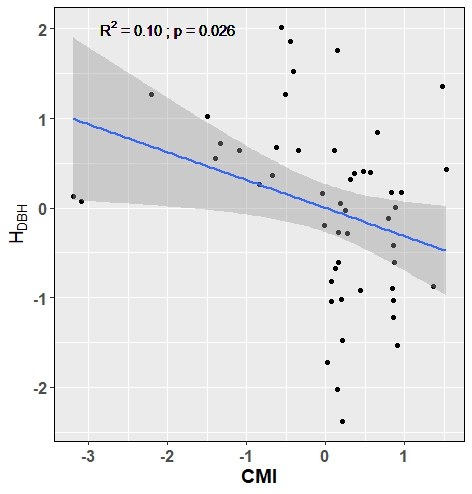 | 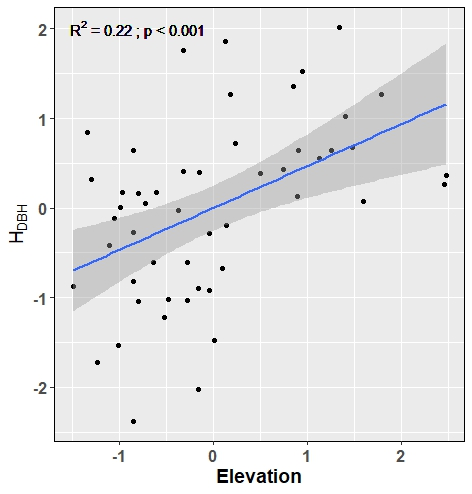 | 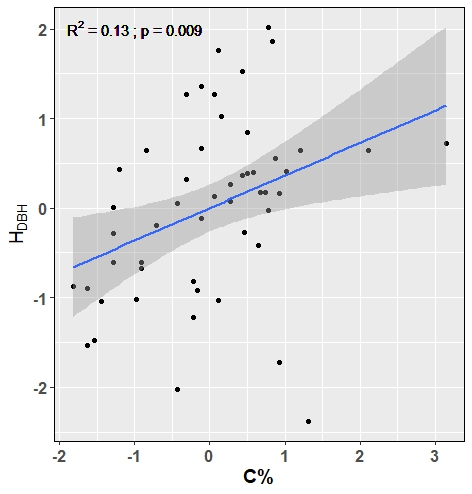 |
| 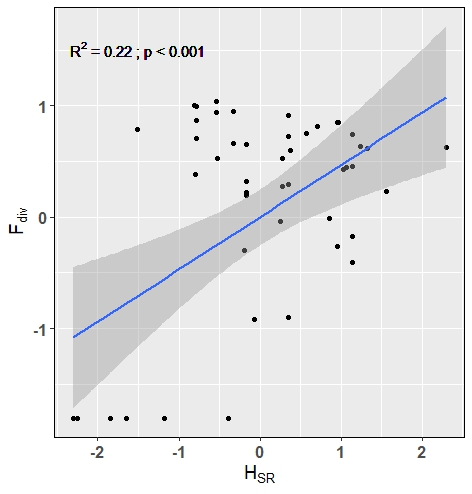 | 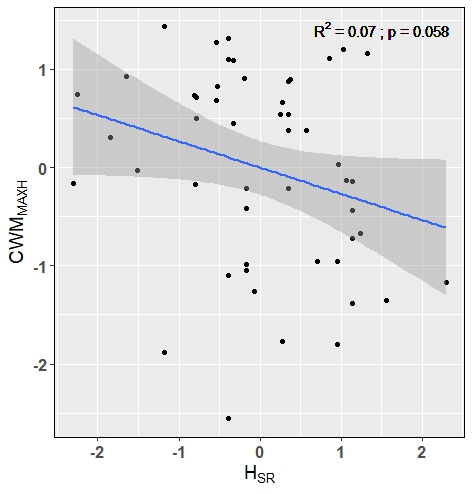 | 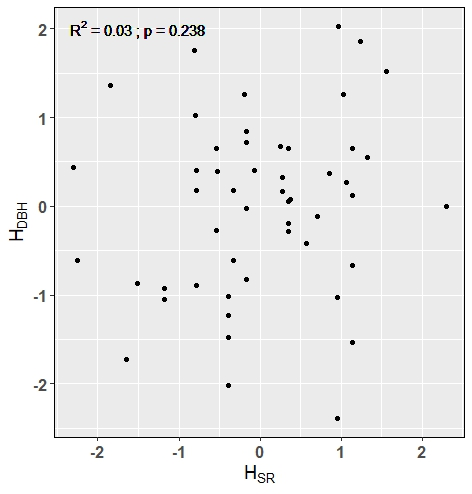 | 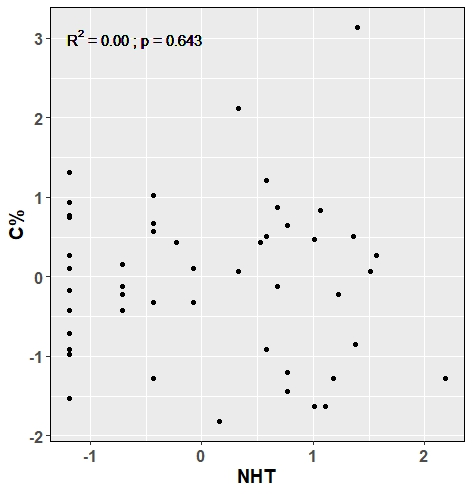 |

**Fig. S4.** Bivariate relationships between all the variables included in the piecewise structural equation model (pSEM; Fig. 4). AGC, NHT, elevation and H_SR_ were log-transformed, whereas C% was square root-transformed. The standardized values were used to plot the bivariate relationships. Linear fitted lines and their pointwise 95% confidence intervals (shaded areas around the lines) are shown for the significant bivariate relationships only. The coefficient of determination (R^2^) and the p-value of the bivariate relationships are also displayed. Acronyms are described in Table S1.

| 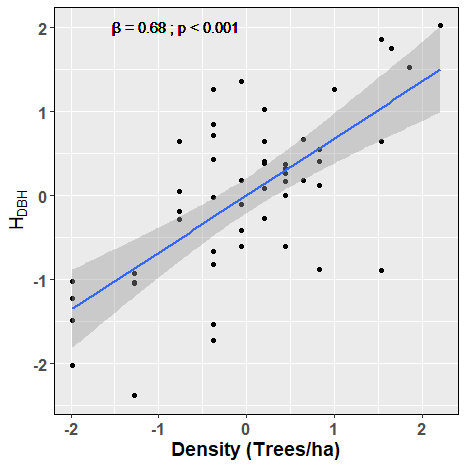(a) | (b) 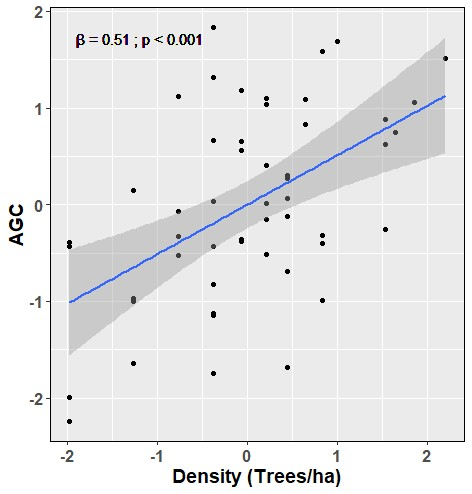 | (c) 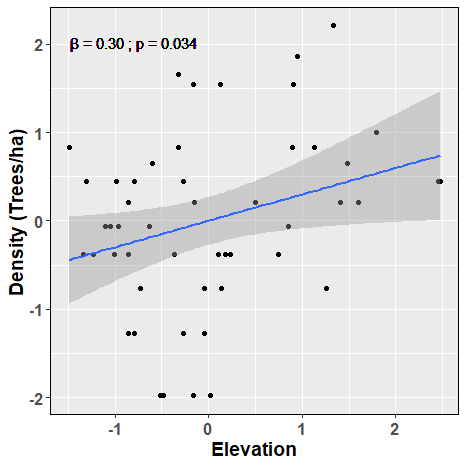 |
| --- | --- | --- |

**Fig. S5.** Bivariate relationships between H_DBH_ and stem density (a), between AGC and stem density (b), and between stem density and elevation (c). Elevation, stem density and AGC were log-transformed. The standardized values were used to plot the bivariate relationships. Linear fitted lines and their pointwise 95% confidence intervals (shaded areas around the lines) are shown. The slope (β) and the p-value of the bivariate relationships are also displayed. Acronyms are described in Table S1.

**References**

Bayen, P., Noulèkoun, F., Bognounou, F., Lykke, A.M., Djomo, A., Lamers, J.P.A., Thiombiano, A., 2020. Models for estimating aboveground biomass of four dryland woody species in Burkina Faso, West Africa. Journal of Arid Environments 180, 104205. https://doi.org/10.1016/j.jaridenv.2020.104205

Curtis, J.T., McIntosh, R.P., 1951. An Upland Forest Continuum in the Prairie-Forest Border Region of Wisconsin. Ecology 32, 476–496. https://doi.org/10.2307/1931725

Fern, K., 2014. Useful Tropical Plants Database 2014 [WWW Document]. URL https://www.feedipedia.org/node/21211 (accessed 5.23.20).

Hisano, M., Chen, H.Y.H., 2020. Spatial variation in climate modifies effects of functional diversity on biomass dynamics in natural forests across Canada. Glob. Ecol. Biogeogr. 29, 682–695. https://doi.org/10.1111/geb.13060

Hsieh, T.C., Ma, K.H., Chao, A., 2016. iNEXT: an R package for rarefaction and extrapolation of species diversity (Hill numbers). Methods Ecol. Evol. 7, 1451–1456. https://doi.org/10.1111/2041-210X.12613

Jost, L., 2006. Entropy and diversity. Oikos 113, 363–375. https://doi.org/10.1111/j.2006.0030-1299.14714.x

Laliberté, E., Legendre, P., 2010. A distance-based framework for measuring functional diversity from multiple traits. Ecology 91, 299–305. https://doi.org/10.1890/08-2244.1

Laliberté, E., Legendre, P., Shipley, B., 2015. Measuring functional diversity (FD) from multiple traits, and other tools for functional ecology. R package version 1.0-12. 1–28.

Lavorel, S., Grigulis, K., McIntyre, S., Williams, N.S.G., Garden, D., Dorrough, J., Berman, S., Quétier, F., Thébault, A., Bonis, A., 2008. Assessing functional diversity in the field – methodology matters! Functional Ecology 22, 134–147. https://doi.org/10.1111/j.1365-2435.2007.01339.x

Staudhammer, C.L., LeMay, V.M., 2011. Introduction and evaluation of possible indices of stand structural diversity. Can. J. For. Res. https://doi.org/10.1139/x01-033

Tadesse Kifle, E., Noulèkoun, F., Son, Y., Khamzina, A., 2022. Woody species diversity, structural composition, and human use of church forests in central Ethiopia. Forest Ecology and Management 506, 119991. https://doi.org/10.1016/j.foreco.2021.119991
