# Supplementary figures and images for: Tree size diversity is the major driver of aboveground carbon storage in dryland agroforestry parklands
Source: Sci Rep. 2023 Dec 14;13:22210. doi: 10.1038/s41598-023-49119-9 (PMC10721610; doi:10.1038/s41598-023-49119-9)

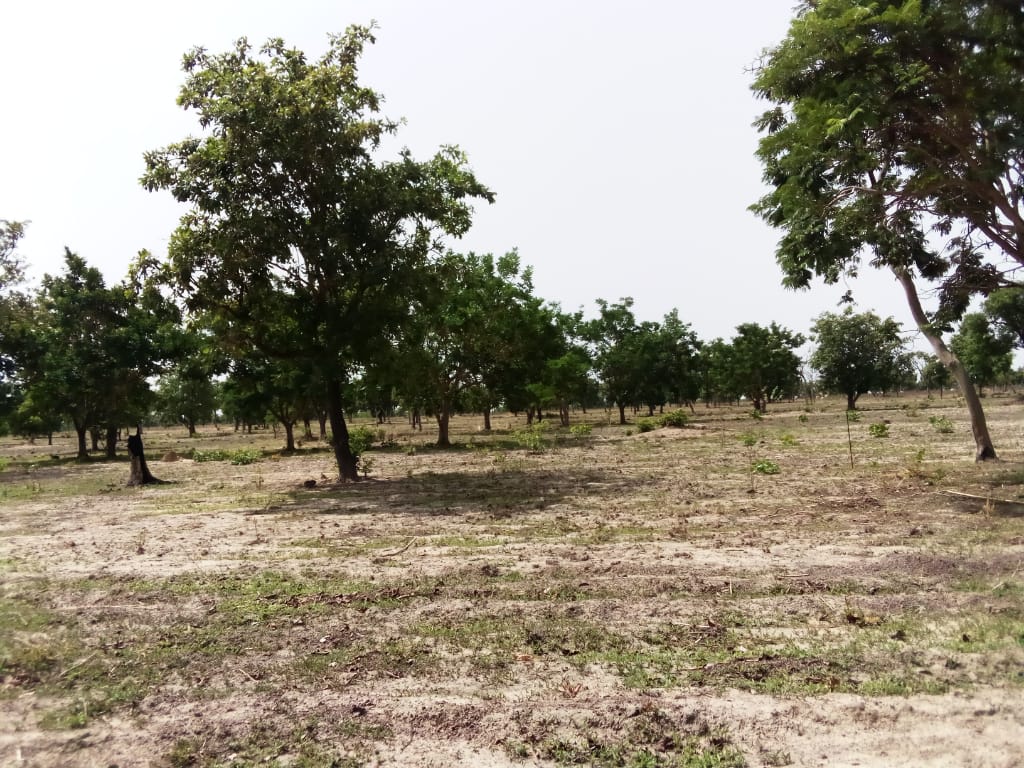

Supplement: Supplementary file 2 — Supplementary Figure S1. [file 41598_2023_49119_MOESM2_ESM.zip › Fig. S1/Fig. S1_left.jpeg]

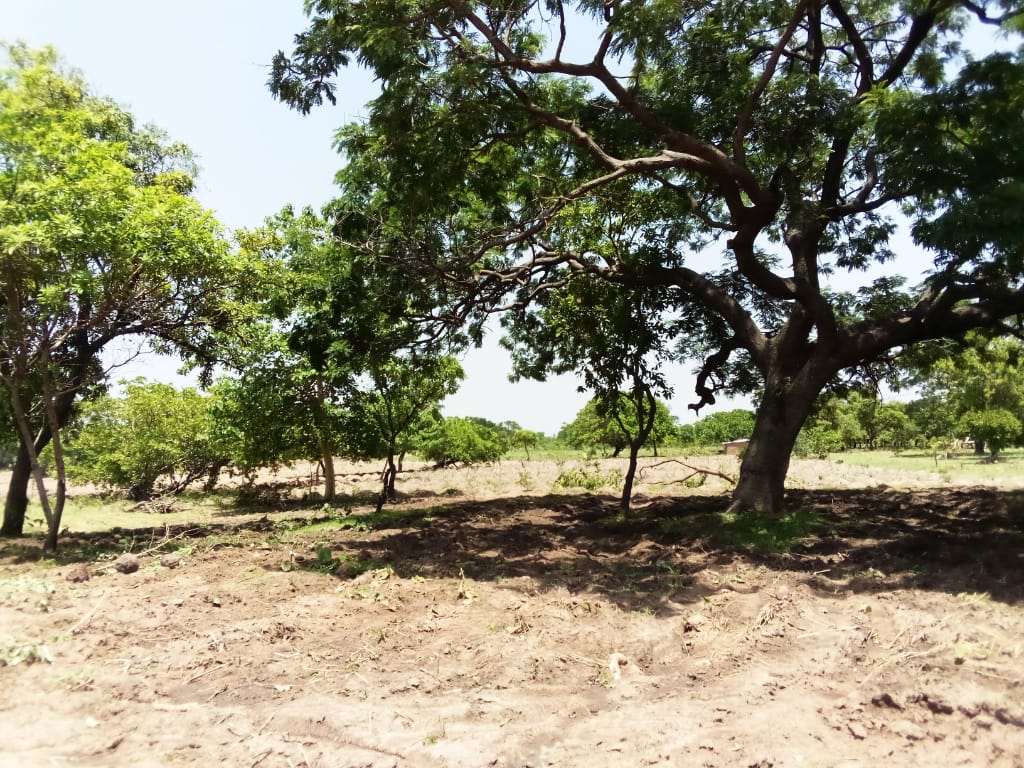

Supplement: Supplementary file 2 — Supplementary Figure S1. [file 41598_2023_49119_MOESM2_ESM.zip › Fig. S1/Fig. S1_right.jpeg]

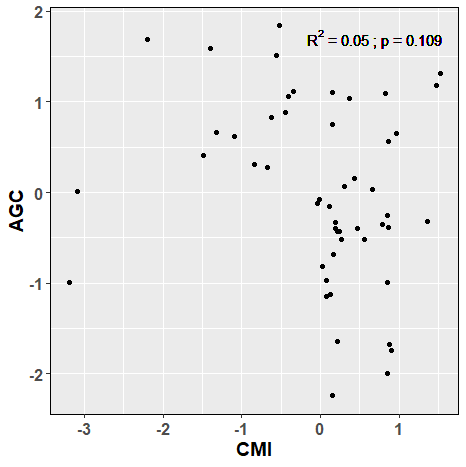

Supplement: Supplementary file 3 — Supplementary Figure S4. [file 41598_2023_49119_MOESM3_ESM.zip › Fig. S4/Fig. S4_ AGC vs CMI.png]

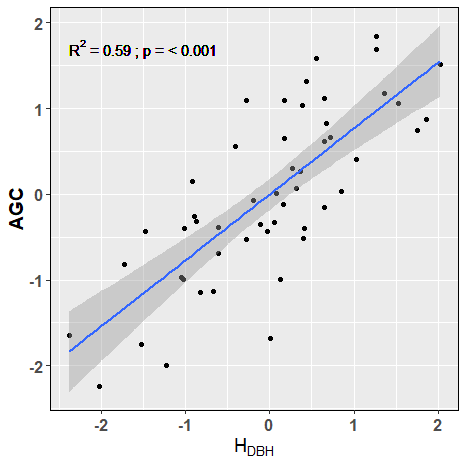

Supplement: Supplementary file 3 — Supplementary Figure S4. [file 41598_2023_49119_MOESM3_ESM.zip › Fig. S4/Fig. S4_ AGC vs HDBH.png]

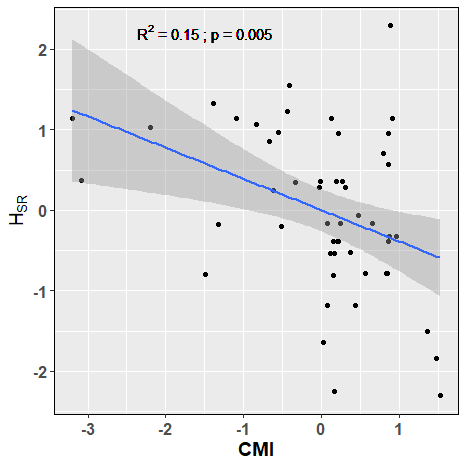

Supplement: Supplementary file 3 — Supplementary Figure S4. [file 41598_2023_49119_MOESM3_ESM.zip › Fig. S4/Fig. S4_ HSR vs CMI.png]

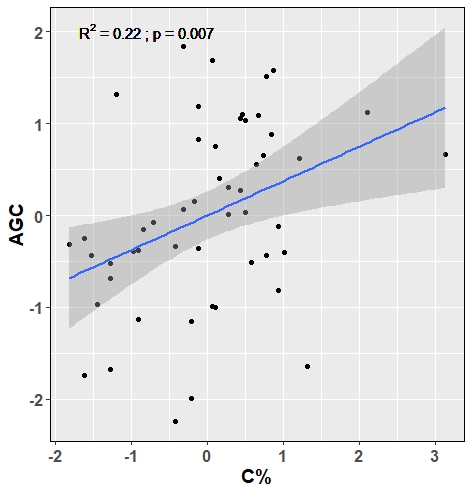

Supplement: Supplementary file 3 — Supplementary Figure S4. [file 41598_2023_49119_MOESM3_ESM.zip › Fig. S4/Fig. S4_AGC vs C%.jpeg]

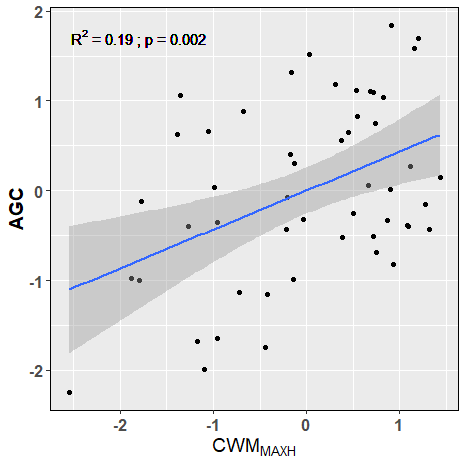

Supplement: Supplementary file 3 — Supplementary Figure S4. [file 41598_2023_49119_MOESM3_ESM.zip › Fig. S4/Fig. S4_AGC vs CWMmaxh.png]

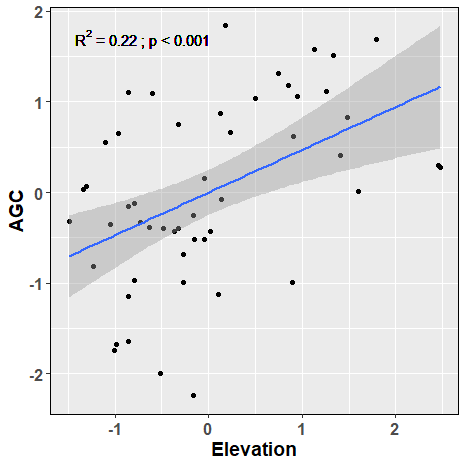

Supplement: Supplementary file 3 — Supplementary Figure S4. [file 41598_2023_49119_MOESM3_ESM.zip › Fig. S4/Fig. S4_AGC vs Elevation.png]

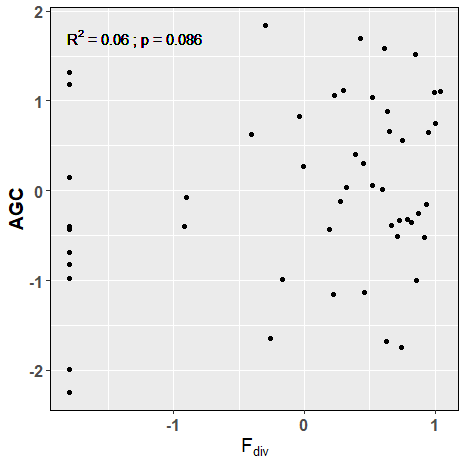

Supplement: Supplementary file 3 — Supplementary Figure S4. [file 41598_2023_49119_MOESM3_ESM.zip › Fig. S4/Fig. S4_AGC vs Fdiv.png]

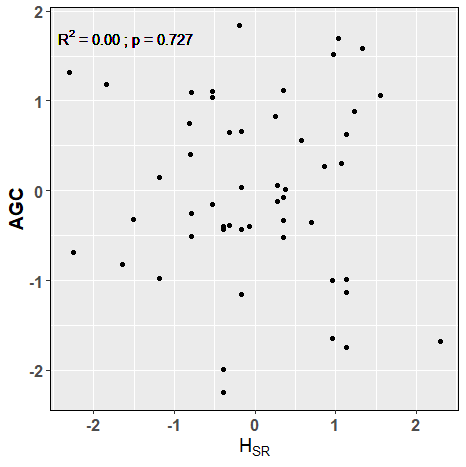

Supplement: Supplementary file 3 — Supplementary Figure S4. [file 41598_2023_49119_MOESM3_ESM.zip › Fig. S4/Fig. S4_AGC vs HSR.png]

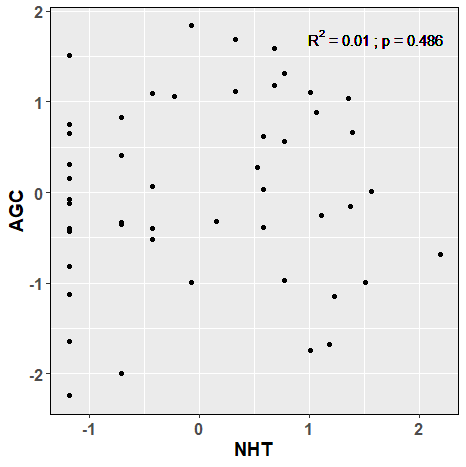

Supplement: Supplementary file 3 — Supplementary Figure S4. [file 41598_2023_49119_MOESM3_ESM.zip › Fig. S4/Fig. S4_AGC vs NHT.png]

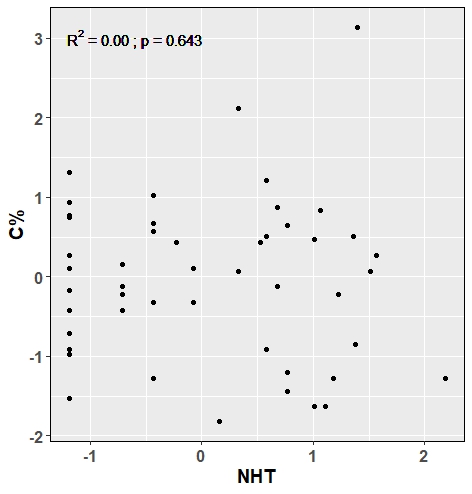

Supplement: Supplementary file 3 — Supplementary Figure S4. [file 41598_2023_49119_MOESM3_ESM.zip › Fig. S4/Fig. S4_C% vs NHT.jpeg]

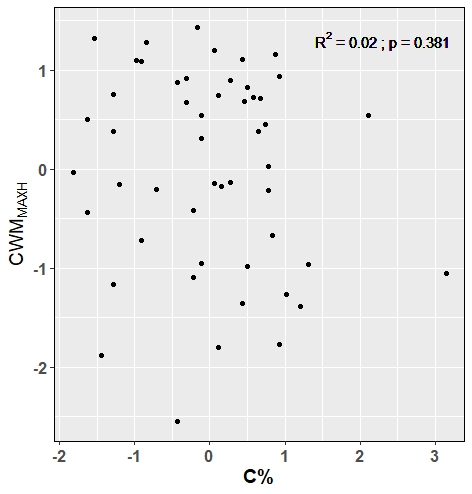

Supplement: Supplementary file 3 — Supplementary Figure S4. [file 41598_2023_49119_MOESM3_ESM.zip › Fig. S4/Fig. S4_CWMmaxh vs C%.jpeg]

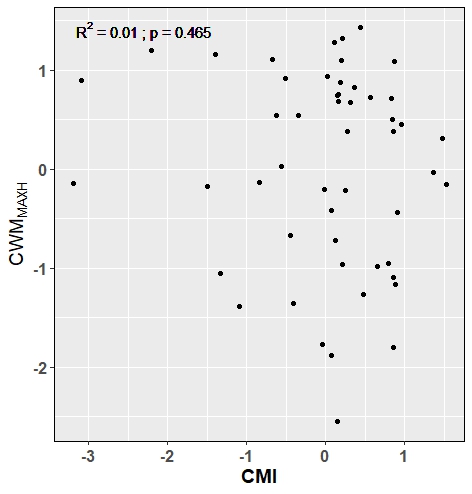

Supplement: Supplementary file 3 — Supplementary Figure S4. [file 41598_2023_49119_MOESM3_ESM.zip › Fig. S4/Fig. S4_CWMmaxh vs CMI.jpeg]

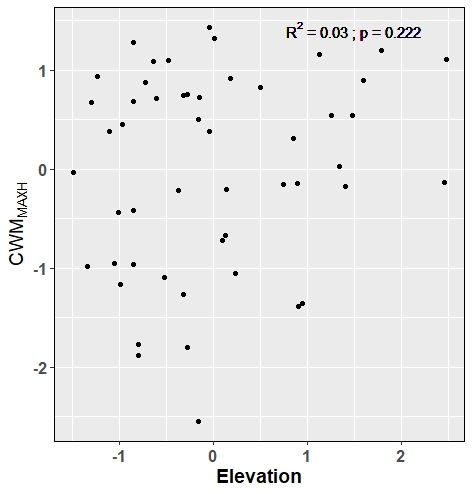

Supplement: Supplementary file 3 — Supplementary Figure S4. [file 41598_2023_49119_MOESM3_ESM.zip › Fig. S4/Fig. S4_CWMmaxh vs Elevation.jpeg]

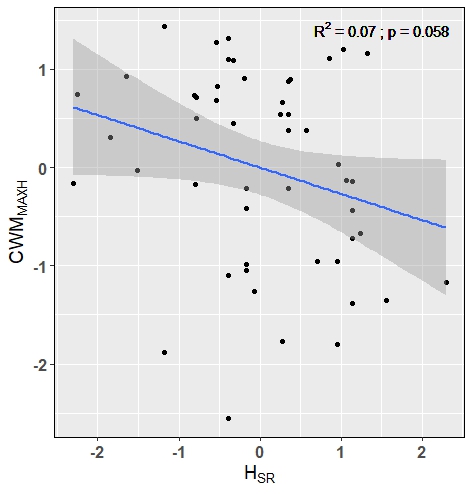

Supplement: Supplementary file 3 — Supplementary Figure S4. [file 41598_2023_49119_MOESM3_ESM.zip › Fig. S4/Fig. S4_CWMmaxh vs HSR.jpeg]

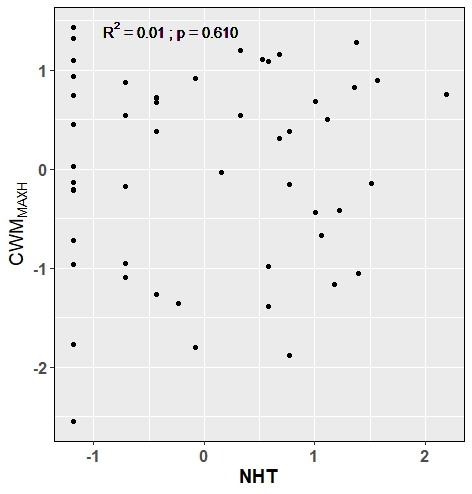

Supplement: Supplementary file 3 — Supplementary Figure S4. [file 41598_2023_49119_MOESM3_ESM.zip › Fig. S4/Fig. S4_CWMmaxh vs NHT.jpeg]

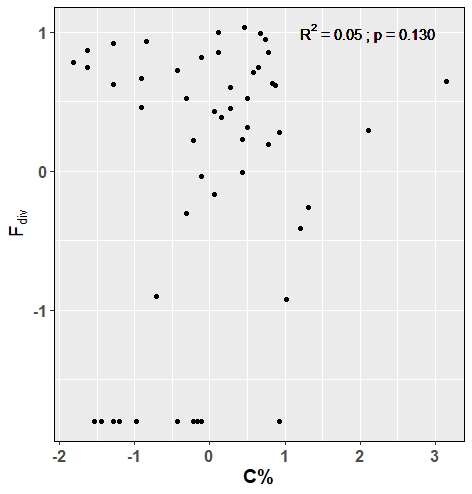

Supplement: Supplementary file 3 — Supplementary Figure S4. [file 41598_2023_49119_MOESM3_ESM.zip › Fig. S4/Fig. S4_Fdiv vs C%.jpeg]

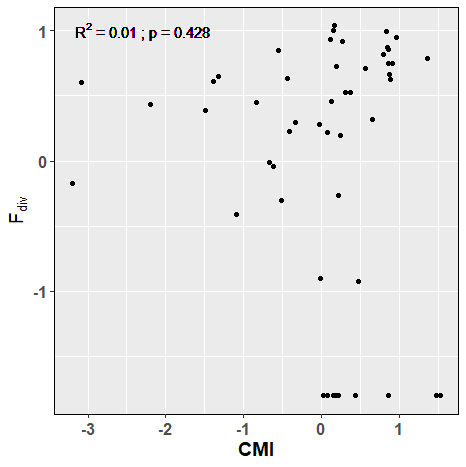

Supplement: Supplementary file 3 — Supplementary Figure S4. [file 41598_2023_49119_MOESM3_ESM.zip › Fig. S4/Fig. S4_Fdiv vs CMI.png]

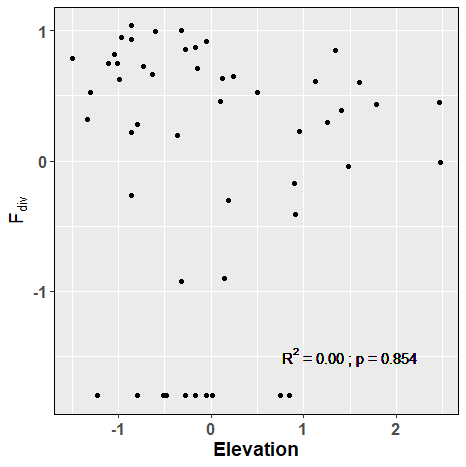

Supplement: Supplementary file 3 — Supplementary Figure S4. [file 41598_2023_49119_MOESM3_ESM.zip › Fig. S4/Fig. S4_Fdiv vs Elevation.png]

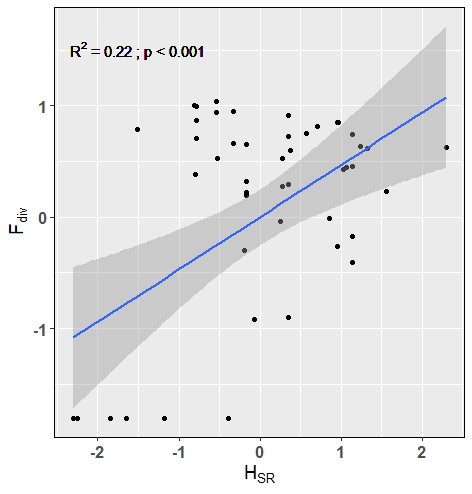

Supplement: Supplementary file 3 — Supplementary Figure S4. [file 41598_2023_49119_MOESM3_ESM.zip › Fig. S4/Fig. S4_Fdiv vs HSR.jpeg]

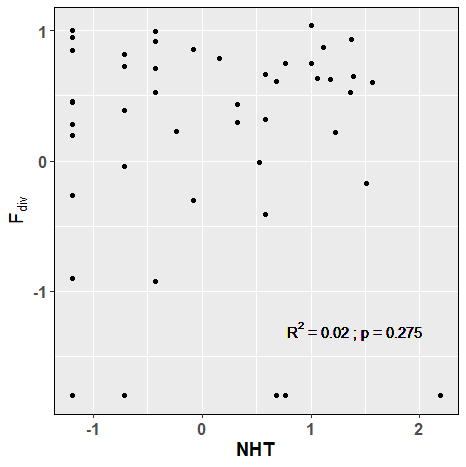

Supplement: Supplementary file 3 — Supplementary Figure S4. [file 41598_2023_49119_MOESM3_ESM.zip › Fig. S4/Fig. S4_Fdiv vs NHT.png]

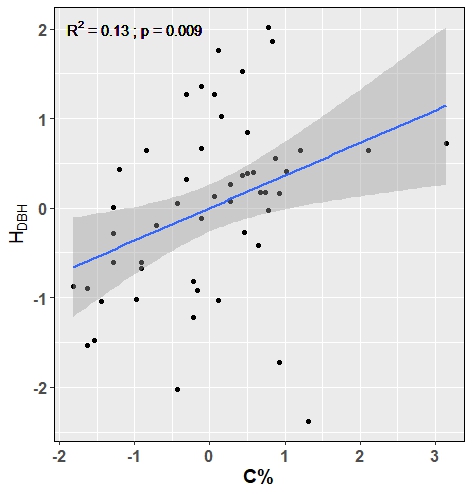

Supplement: Supplementary file 3 — Supplementary Figure S4. [file 41598_2023_49119_MOESM3_ESM.zip › Fig. S4/Fig. S4_HDBH vs C%.jpeg]

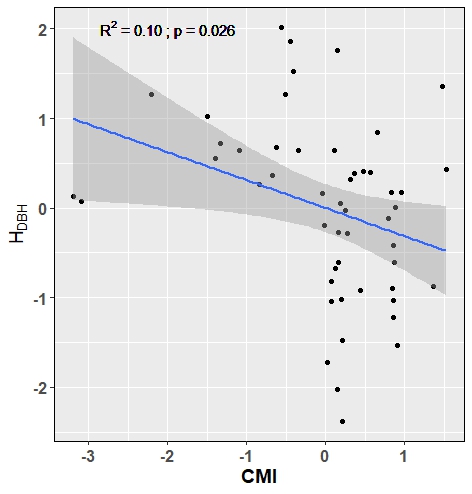

Supplement: Supplementary file 3 — Supplementary Figure S4. [file 41598_2023_49119_MOESM3_ESM.zip › Fig. S4/Fig. S4_HDBH vs CMI.jpeg]

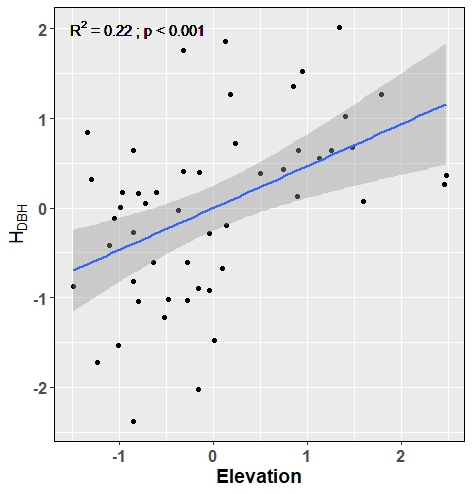

Supplement: Supplementary file 3 — Supplementary Figure S4. [file 41598_2023_49119_MOESM3_ESM.zip › Fig. S4/Fig. S4_HDBH vs Elevation.jpeg]

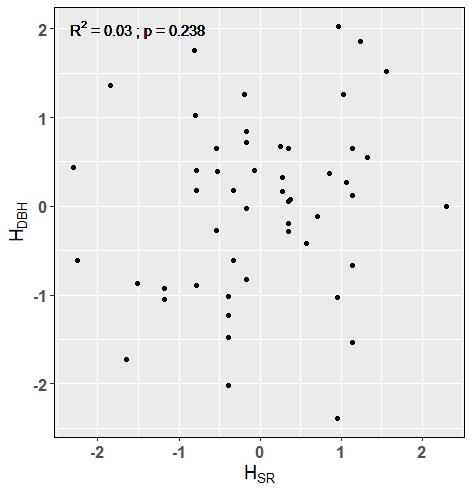

Supplement: Supplementary file 3 — Supplementary Figure S4. [file 41598_2023_49119_MOESM3_ESM.zip › Fig. S4/Fig. S4_HDBH vs HSR.jpeg]

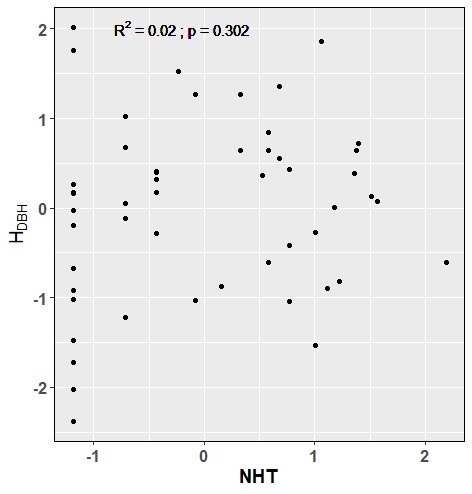

Supplement: Supplementary file 3 — Supplementary Figure S4. [file 41598_2023_49119_MOESM3_ESM.zip › Fig. S4/Fig. S4_HDBH vs NHT.jpeg]

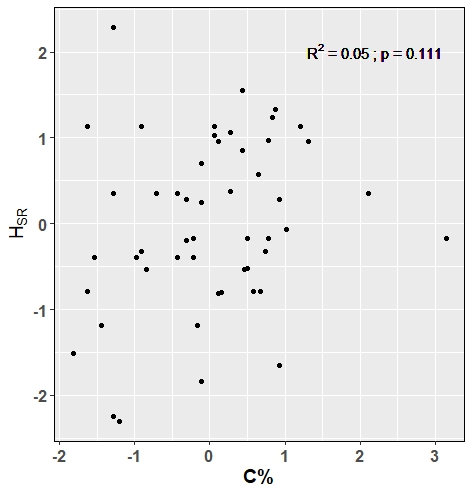

Supplement: Supplementary file 3 — Supplementary Figure S4. [file 41598_2023_49119_MOESM3_ESM.zip › Fig. S4/Fig. S4_HSR vs C%.jpeg]

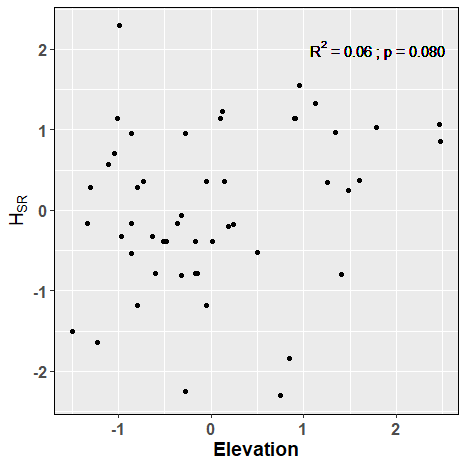

Supplement: Supplementary file 3 — Supplementary Figure S4. [file 41598_2023_49119_MOESM3_ESM.zip › Fig. S4/Fig. S4_HSR vs Elevation.png]

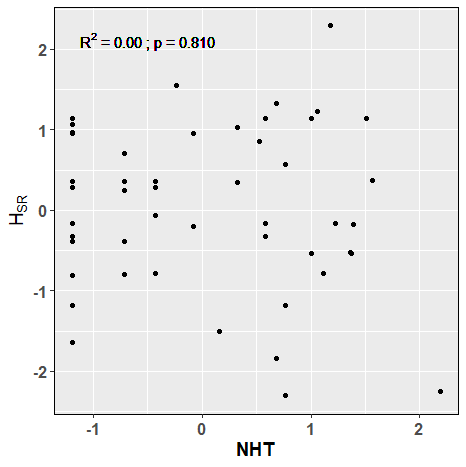

Supplement: Supplementary file 3 — Supplementary Figure S4. [file 41598_2023_49119_MOESM3_ESM.zip › Fig. S4/Fig. S4_HSR vs NHT.png]

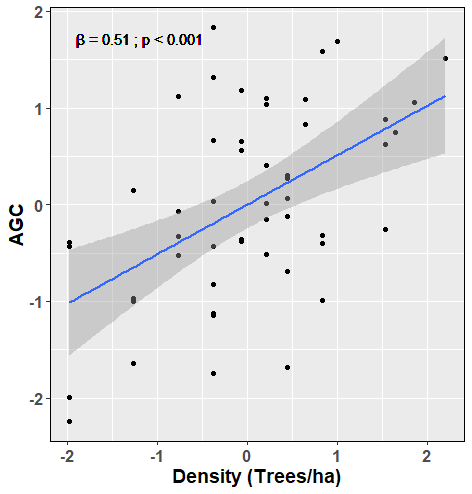

Supplement: Supplementary file 4 — Supplementary Figure S5. [file 41598_2023_49119_MOESM4_ESM.zip › Fig. S5/Fig. S5_AGC vs Density.png]

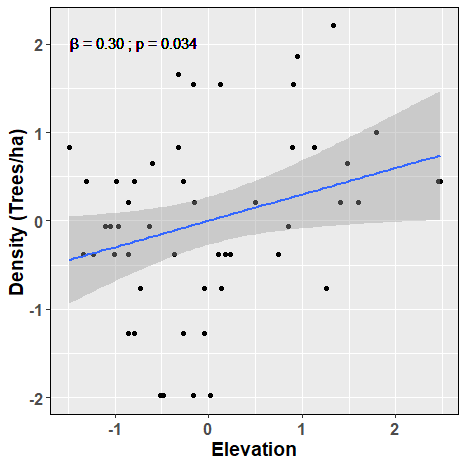

Supplement: Supplementary file 4 — Supplementary Figure S5. [file 41598_2023_49119_MOESM4_ESM.zip › Fig. S5/Fig. S5_Density vs Elevation.png]

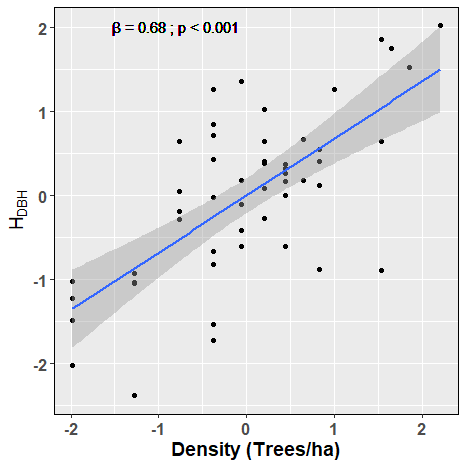

Supplement: Supplementary file 4 — Supplementary Figure S5. [file 41598_2023_49119_MOESM4_ESM.zip › Fig. S5/Fig. S5_HDBH vs Density.png]
